# SIFamide-dependent synaptic plasticity in male-specific GABAergic neurons underlies experience-modulated mating behaviors

**DOI:** 10.1016/j.isci.2025.113516

**Published:** 2025-09-05

**Authors:** Tianmu Zhang, Hongyu Miao, Yutong Song, Zekun Wu, Woo Jae Kim

**Affiliations:** 1 HIT Center for Life Sciences, School of Life Science and Technology, Harbin Institute of Technology, Harbin 150001, China; 2 Medical and Health Research Institute, Zhengzhou Research Institute of HIT, Zhengzhou, Henan 450000, China

**Keywords:** Biological sciences, Neuroscience, Behavioral neuroscience

## Abstract

*Drosophila melanogaster* relies on complex brain circuits and neuromodulation for experience-dependent sexual behavior, yet the role of neuropeptide signaling in behavioral refinement remains unclear. We investigate SIFamide (SIFa) and its receptor (SIFaR) in regulating male *Drosophila* sexual behavior. SIFaR is essential for these behaviors, particularly interval timing. Knockdown in fru-positive neurons impairs courtship index (CI) and mating duration (MD) but not copulation latency (CL). Critically, SIFaR in GABAergic mAL neurons (fru^+^, dsx^−^) specifically controls MD and CI, not CL. Additionally, SIFa signaling in mAL neurons of experienced males increases presynaptic terminals, indicating context-dependent synaptic plasticity. These results reveal a complex SIFa/SIFaR interaction with specific neurons in modulating male behavior. The distinct roles of SIFaR in fru-positive versus other neurons, alongside its plasticity function, provide new insights into neural mechanisms of context-dependent behavioral adaptation.

## Highlights

• SIFaR in fru-positive neurons regulates male sexual behavior in *Drosophila*
• mAL GABAergic neurons mediate SIFaR signaling and plasticity by sexual experience
• Sexual experience enhances synaptic connections in fru^+^ mAL neurons
• SIFa signaling acts through both fru-positive and fru-negative neurons

## Graphical abstract

**Figure:**
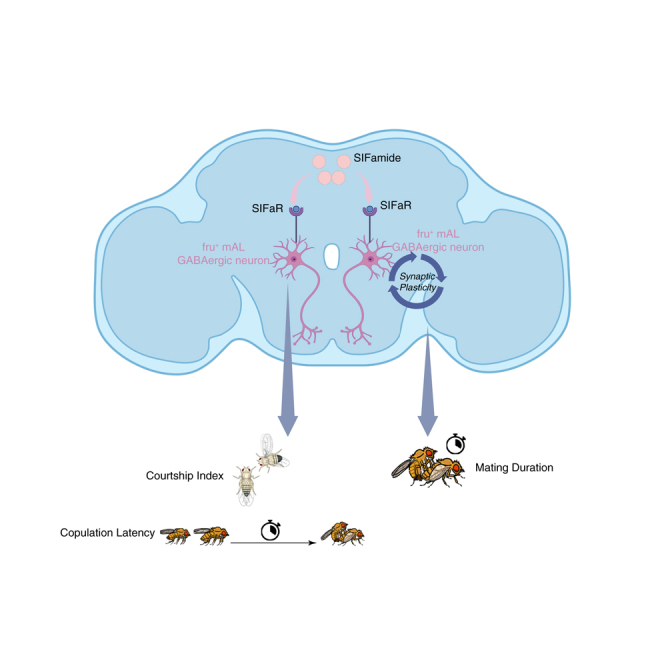

## Introduction

Neuropeptides (NPs) participate in a variety of functions, including hormone control, metabolic processes, and behavior.^1^ Neuropeptides have a significant role in sexual dimorphism, the phenomenon in which the sexes of the same animal exhibit unique physical and/or behavioral characteristics. In certain species, the expression of specific neuropeptides varies between sexes, which may result in distinct behavioral or physical features. For example, corticotropin-releasing factor (CRF) is a neuropeptide that is involved in the regulation of stress and fear responses, and it is present at different concentrations in males and females. In males, CRF is involved in the regulation of aggression, while in females it is involved in the regulation of maternal behavior.^2^^,^^3^

Neuropeptide receptors (NPRs) with sexually dimorphic activity can be found in many different types of organisms, including mammals. The GnRH receptor is an example of a neuropeptide receptor that is expressed differently in males and females of the same species. This receptor is more common in males than in females, and it is thought to play a role in how reproductive physiology and behavior are controlled.^4^^,^^5^ The corazonin receptor (CrzR) in *Drosophila* serves as a notable example of a sexually dimorphic NPR. This receptor exhibits higher expression levels in males compared to females and is believed to play a crucial role in regulating male-specific behaviors, including courtship and aggression.^6^^,^^7^

SIFamide (SIFa) is a neuropeptide expressed in a small group of neurons in the posterior-interior (PI) cluster of the central brain of the fruit fly *Drosophila melanogaster*.^8^ SIFa regulates multiple behaviors, including sleep, feeding, and reproduction. The SIFa receptor (SIFaR) is known to regulate behaviors such as circadian rhythms, food intake, and metabolic processes. It is also known to play a role in the regulation of neuronal communication and excitability, with studies indicating that it has a role in modulating the activity of some neurotransmitters, such as dopamine and serotonin, as well as in influencing the release of hormones. Additionally, SIFaR is thought to be involved in the regulation of complex behaviors such as learning, memory, and decision making.^9^^,^^10^^,^^11^^,^^12^^,^^13^^,^^14^^,^^15^

Emerging evidence suggests that SIFaR-expressing neurons, rather than SIFa-producing neurons, may contribute to the development of sex-specific circuitry within *fru*-defined neuronal populations.^15^ SIFaR knockdown in *fru*-positive cells led to male-male courtship.^16^ Although the potential association between SIFaR signaling and *fru*-positive sexually dimorphic circuits is identified, the intricacies of sexually dimorphic circuits linked to specific behavior have not yet been described. Our previous research has established that the activity of SIFa and the synaptic plasticity resulting from the extensive branching of SIFa neurons are vital for regulating energy balance behaviors in male *Drosophila*, mediated by long-range neuropeptide signaling through SIFaR.^17^^,^^18^

In this work, we reveal that SIFa inputs to SIFaR receptors present in *fru*-expressing mAL neurons are essential for the modulation of energy balance behaviors driven by SIFa. We hypothesize that SIFa inputs play a key role in orchestrating the activity of *fru*-positive pC1 and mAL neurons via SIFaR expression, thereby enabling the processing of information necessary for adjusting internal states related to various energy balance behaviors. This study builds on our earlier findings, highlighting the importance of SIFa signaling in modulating energy balance behaviors through its interactions with SIFaR in specific neuronal populations, particularly those expressing *fruitless*.

## Results

### Diverse input-output combinations of SIFamide neurons regulate male sexual behavior repertoire

**Figure 1:**
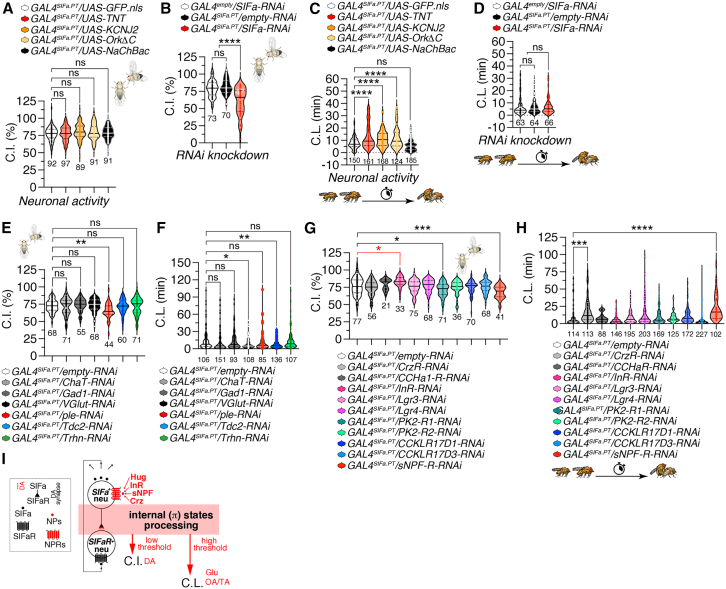
SIFa neuron activity and SIFa levels influence male sexual behavior differently (A) Courtship index (CI) of group-housed males expressing *GAL4*^*SIFa.PT*^ driver together with *UAS-GFP.nls, UAS-TNT, UAS-KCNJ2, UAS-OrkΔC* and *UAS-NaChBac*. CI was measured by [courtship time/(mating time-courtship time) ∗100%]. For detailed methods, see the STAR Methods for a detailed description of the CI assays used in this study. Statistical significance determined by one-way ANOVA followed by Tukey’s multiple comparisons test. *p* = 0.2167 (one-way ANOVA, F = 1.45, R^2^ = 0.01; Tukey’s post hoc). The ns represents non-significant differences. Sample sizes (n) are indicated in the figure panels. Data are represented as mean ± SEM. (B) CI of group-housed males expressing *GAL4*^*SIFa.PT*^ driver together with *SIFa-RNAi*. Flies expressing *GAL4*^*empty*^ with *SIFa-RNAi* and *empty-RNAi* with *SIFa-RNAi* as control. Statistical significance determined by one-way ANOVA followed by Tukey’s multiple comparisons test. *p* < 0.0001 (one-way ANOVA, F = 37.03, R^2^ = 0.28; Tukey’s post hoc). The ns represents non-significant differences. Sample sizes (n) are indicated in the figure panels. Data are represented as mean ± SEM. (C) Copulation latency (CL) of group-housed males expressing *GAL4*^*SIFa.PT*^ driver together with *UAS-GFP.nls, UAS-TNT, UAS-KCNJ2, UAS-OrkΔC* and *UAS-NaChBac*. Statistical significance determined by one-way ANOVA followed by Tukey’s multiple comparisons test. *p* < 0.0001 (one-way ANOVA, F = 29.49, R^2^ = 0.13; Tukey’s post hoc). The ns represents non-significant differences. Sample sizes (n) are indicated in the figure panels. For detailed methods, see the STAR Methods for a detailed description of the CL assays used in this study. Data are represented as mean ± SEM. (D) CL of group-housed males expressing *GAL4*^*SIFa.PT*^ driver together with *SIFa-RNAi*. Flies expressing *GAL4*^*empty*^ with *SIFa-RNAi* and *empty-RNAi* with *SIFa-RNAi* as control. Statistical significance determined by one-way ANOVA followed by Tukey’s multiple comparisons test. *p* = 0.9779 (one-way ANOVA, F = 0.02, R^2^ = 0.00023; Tukey’s post hoc). The ns represents non-significant differences. Sample sizes (n) are indicated in the figure panels. Data are represented as mean ± SEM. (E) CI of group-housed males expressing *GAL4*^*SIFa.PT*^ driver together with *empty-RNAi, ChaT-RNAi, Gad1-RNAi, VGlut-RNAi*, *ple-RNAi, Tdc2-RNAi and Trhn-RNAi*. Statistical significance determined by one-way ANOVA followed by Tukey’s multiple comparisons test. *p* < 0.0001 (one-way ANOVA, F = 8.69, R^2^ = 0.11; Tukey’s post hoc). The ns represents non-significant differences. Sample sizes (n) are indicated in the figure panels. Data are represented as mean ± SEM. (F) CL of group-housed males expressing *GAL4*^*SIFa.PT*^ driver together with *empty-RNAi, ChaT-RNAi, Gad1-RNAi, VGlut-RNAi*, *ple-RNAi, Tdc2-RNAi and Trhn-RNAi*. Statistical significance determined by one-way ANOVA followed by Tukey’s multiple comparisons test. *p* = 0.4548 (one-way ANOVA, F = 0.96, R^2^ = 0.0085; Tukey’s post hoc). The ns represents non-significant differences. Sample sizes (n) are indicated in the figure panels. Data are represented as mean ± SEM. (G) CI of group-housed males expressing *GAL4*^*SIFa.PT*^ driver together with *empty-RNAi, CrzR-RNAi, CCHa1-R-RNAi, InR-RNAi, Lgr3-RNAi, Lgr4-RNAi, Pk2-R1-RNAi, PK2-R2-RNAi, CCKLR17D1-RNAi, CCKLR17D3-RNAi* and *sNPF-R-RNAi*. Statistical significance determined by one-way ANOVA followed by Tukey’s multiple comparisons test. *p* < 0.0001 (one-way ANOVA, F = 5.66, R^2^ = 0.086; Tukey’s post hoc). The ns represents non-significant differences. Sample sizes (n) are indicated in the figure panels. Data are represented as mean ± SEM. (H) CL of group-housed males expressing *GAL4*^*SIFa.PT*^ driver together with *empty-RNAi, CrzR-RNAi, CCHa1-R-RNAi, InR-RNAi, Lgr3-RNAi, Lgr4-RNAi, Pk2-R1-RNAi, PK2-R2-RNAi, CCKLR17D1-RNAi, CCKLR17D3-RNAi* and *sNPF-R-RNAi*. Statistical significance determined by one-way ANOVA followed by Tukey’s multiple comparisons test. *p* < 0.0001 (one-way ANOVA, F = 25.55, R^2^ = 0.13; Tukey’s post hoc). The ns represents non-significant differences. Sample sizes (n) are indicated in the figure panels. Data are represented as mean ± SEM. (I) Schematic representation of SIFa neurons and SIFaR neurons regulating internal states.

SIFa has been recognized as a multifunctional peptide, but it was first identified for its involvement in male-male courtship behavior.^15^ Research indicates that the removal of SIFa-expressing neurons or the reduction of SIFa peptide levels can lead to an increase in male-to-male courtship behaviors.^15^ To gain insights into the mechanisms by which SIFa neurons modulate sexual behaviors, we initially attempted to alter neuronal activity in SIFa-positive neurons using various activity modifiers under the *GAL4*^*SIFa.PT*^ driver. Notably, expressing TNT (tetanus toxin light chain), potassium channels KCNJ2/OrkΔC,^19^ or the bacterial sodium channel NaChBac within SIFa neurons did not significantly affect courtship indices when compared to control flies (Figure 1A). Conversely, a reduction in SIFa peptide levels via RNA interference (RNAi) led to a substantial decrease in male courtship indices toward females compared to control groups (Figure 1B).

The use of TNT has proven effective in inhibiting neurotransmitter release across several studies; KCNJ2/OrkΔC prevents membrane depolarization, while NaChBac induces sodium conductance that results in cell depolarization.^20^ Thus, our research indicates that the neuropeptide SIFa’s release is more essential for normal male courtship behavior than the neuronal activity of SIFa-expressing neurons themselves.

Next, we examined another aspect of male sexual behavior: copulation (or mating) latency (CL). CL, defined as the time it takes for a male to initiate copulation with a female, is a critical behavioral trait that can significantly impact reproductive success in various species, including *Drosophila melanogaster*. From an evolutionary perspective, CL can be influenced by several factors, including competition among males and the quality of potential mates. For instance, males exposed to rival males often exhibit shorter mating latencies, suggesting an adaptive response to increased competition.^21^^,^^22^^,^^23^ This phenomenon indicates that males may prioritize rapid mating to maximize reproductive opportunities when faced with competitors. CL reflects an integrated behavioral output influenced by male courtship intensity, female receptivity, and copulatory performance. To specifically dissect male contributions to CL, all assays utilized SPR-deficient virgin females (*SPR*^−/−^), which exhibit constitutively high receptivity and minimal rejection behavior.^24^ This approach standardizes female responsiveness, thereby isolating genetic and neuronal mechanisms underlying male courtship initiation, persistence, and copulation efficiency. Under these controlled conditions, CL serves as a quantifiable proxy for male eagerness and performance.

Inhibiting SIFa neuronal activity by expressing TNT and KCNJ2/OrkΔC led to a notable delay in CL, while the hyperactivation of SIFa neurons through NaChBac did not influence CL (Figure 1C). Furthermore, reducing SIFa levels via RNAi also failed to affect CL (Figure 1D). Unlike the data for CI, these results imply that the active states of SIFa neurons are more essential for sustaining rapid CL than the release of the SIFa neuropeptide. Overall, our findings reveal that CI and CL, two interconnected facets of male sexual behavior, are governed by different neural mechanisms associated with SIFa neurons.

Numerous studies have indicated that SIFa neurons predominantly employ glutamate (Glu) and dopamine (DA) as their main neurotransmitters to regulate sleep and interval timing behaviors.^11^^,^^18^^,^^25^ To ascertain which neurotransmitter system contributes to CI and CL, we conducted RNAi to knock down essential genes involved in the production of each neurotransmitter. We targeted *VGlut-RNAi* (vesicular glutamate transporter)^26^ to impair the glutamatergic (Glu) system, *ple-RNAi* (pale)^27^ for the dopaminergic (DA) system, *Gad1-RNAi* (glutamic acid decarboxylase 1)^28^ for the GABAergic (GABA) system, *ChaT-RNAi* (choline acetyltransferase)^26^ for the cholinergic (Cha) system, *Tdc2-RNAi* (tyrosine decarboxylase 2)^29^ for the octopaminergic/tyraminergic (OA/TA) system and *Trhn-RNAi* (Tryptophan hydroxylase neuronal)^26^ for the serotoninergic (5-HT) system. Our findings from RNAi-mediated knockdown experiments indicate that the DA system is essential for facilitating normal CI behavior in SIFa-positive neurons (Figure 1E). Conversely, our results suggest that CL is largely dependent of Glu and OA/TA systems produced by SIFa neurons (Figure 1F). These observations imply that while CI is dependent on SIFa and DA transmission, CL may function independently through Glu and OA/TA synaptic transmission mechanisms instead.^30^

The SIFa neurons are known to integrate signals from various peptidergic pathways such as Crz, dilp2, Dsk, sNPF, MIP, and hugin.^1^^,^^14^^,^^31^ To determine which neuropeptide inputs are crucial for CI and CL, we conducted a systematic analysis of each neuropeptide receptor’s role within SIFa-positive cells. A reduction in InR levels within SIFa neurons led to a notable increase in CI compared to control flies. In contrast, the knockdown of PK2-R2 (hugin receptor) and sNPF-R levels resulted in a significant decrease in CI relative to control groups (Figure 1G). Furthermore, reducing CrzR and sNPF-R levels in SIFa neurons caused delays in CL (Figure 1H). These results suggest that sNPF signaling is vital for modulating both CI and CL, while insulin and hugin signaling specifically regulate CI, with Crz signaling exclusively influencing CL (Figures 1G and 1H). Overall, our findings indicate that CI and CL rely on distinct input-output pathways from SIFa neurons for their appropriate generation.^26^^,^^27^^,^^28^

### Male-specific SIFamide receptor-expressing neurons differentially mediate SIFamide signaling to generate internal states for varied sexual behaviors

It is well established that SIFa modulates sexual behavior through its action on *fruitless* neurons. In *Drosophila melanogaster*, the *fruitless* gene encodes male-specific transcription factors that delineate distinct neuronal circuits involved in courtship behavior. Mutations in the *fruitless* gene can lead to bisexual courting behaviors in males, where they exhibit courtship toward both females and other males.^32^ Utilizing RNAi to specifically diminish SIFaR expression within these neurons, the authors showed that this alteration resulted in heightened male-male courtship behaviors.^16^ This evidence underscores the significant role of SIFa signaling through *fruitless* neurons in shaping sexual behaviors in *Drosophila*.

**Figure 2:**
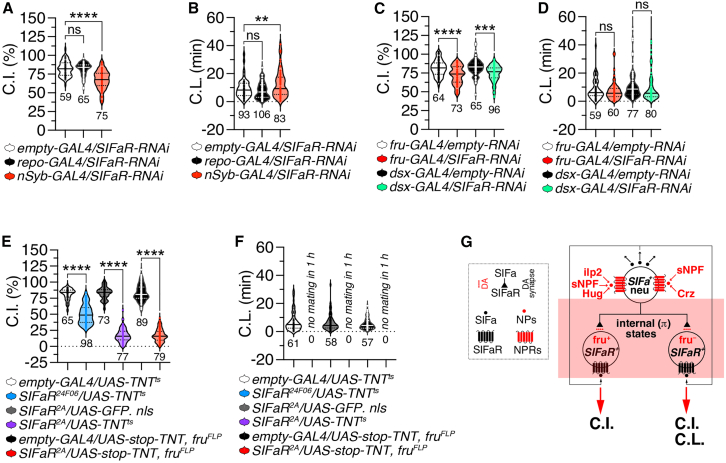
SIFaR expression in male-specific neurons is crucial for male sexual behavior (A) CI of group-housed males expressing *empty-GAL4, repo-GAL4* and *nSyb-GAL4* together with *SIFaR-RNAi*. Statistical significance determined by one-way ANOVA followed by Tukey’s multiple comparisons test. *p* < 0.0001 (one-way ANOVA, F = 43.66, R^2^ = 0.31; Tukey’s post hoc). The ns represents non-significant differences. Sample sizes (n) are indicated in the figure panels. Data are represented as mean ± SEM. (B) CL of group-housed males expressing *empty-GAL4, repo-GAL4* and *nSyb-GAL4* together with *SIFaR-RNAi*. Statistical significance determined by one-way ANOVA followed by Tukey’s multiple comparisons test. *p* < 0.0001 (one-way ANOVA, F = 9.39, R^2^ = 0.064; Tukey’s post hoc). The ns represents non-significant differences. Sample sizes (n) are indicated in the figure panels. Data are represented as mean ± SEM. (C) CI of group-housed males expressing *fru-GAL4* and *dsx-GAL4* driver together with *empty-RNAi* and *SIFaR-RNAi*. Statistical significance determined by one-way ANOVA followed by Tukey’s multiple comparisons test. *p* < 0.0001 (one-way ANOVA, F = 14.82, R^2^ = 0.13; Tukey’s post hoc). The ns represents non-significant differences. Sample sizes (n) are indicated in the figure panels. Data are represented as mean ± SEM. (D) CL of group-housed males expressing *fru-GAL4* and *dsx-GAL4* driver together with *empty-RNAi* and *SIFaR-RNAi*. Statistical significance determined by one-way ANOVA followed by Tukey’s multiple comparisons test. *p* = 0.046 (one-way ANOVA, F = 2.70, R^2^ = 0.029; Tukey’s post hoc). The ns represents non-significant differences. Sample sizes (n) are indicated in the figure panels. Data are represented as mean ± SEM. (E) CI of group-housed males expressing *empty-GAL4* with *UAS-TNT/tub-GAL80*^*ts*^*, SIFaR*^*24F06*^ with *UAS-TNT/tub-GAL80*^*ts*^*, SIFaR*^*2A*^*-GAL4* with *UAS-GFP. nls*, *SIFaR-GAL4* with *UAS-TNT/tub-GAL80*^*ts*^, *empty-GAL4* with *UAS-stop-TNT, fru*^*FLP*^ and *SIFaR*^*2A*^*-GAL4* with *UAS-stop-TNT, fru*^*FLP*^. Statistical significance determined by one-way ANOVA followed by Tukey’s multiple comparisons test. *p* < 0.0001 (one-way ANOVA, F = 584.9, R^2^ = 0.86; Tukey’s post hoc). The ns represents non-significant differences. Sample sizes (n) are indicated in the figure panels. Data are represented as mean ± SEM. (F) CL of group-housed males expressing *empty-GAL4* with *UAS-TNT/tub-GAL80*^*ts*^*, SIFaR*^*24F06*^ with *UAS-TNT/tub-GAL80*^*ts*^*, SIFaR*^*2A*^*-GAL4* with *UAS-GFP. nls*, *SIFaR-GAL4* with *UAS-TNT/tub-GAL80*^*ts*^, *empty-GAL4* with *UAS-stop-TNT, fru*^*FLP*^ and *SIFaR*^*2A*^*-GAL4* with *UAS-stop-TNT, fru*^*FLP*^. Statistical significance determined by one-way ANOVA followed by Tukey’s multiple comparisons test. *p* = 0.3251 (one-way ANOVA, F = 1.16, R^2^ = 0.018; Tukey’s post hoc). The ns represents non-significant differences. Sample sizes (n) are indicated in the figure panels. Data are represented as mean ± SEM. (G) Schematic representation of SIFa neurons regulating courtship index and courtship latency.

Previous research has shown that SIFa inputs specifically target various SIFaR neuronal circuits, which are integral to the modulation of interval timing behaviors.^10^^,^^17^^,^^25^ We suggest that the chain reaction of peptidergic signaling from SIFa to SIFaR circuits establishes context-sensitive internal states, facilitating the regulation of a multifaceted behavioral repertoire (Figure S1A). The reduction of SIFaR levels via RNAi specifically in neurons results in a marked decrease in CI and a delay in CL (Figures 2A and 2B), indicating that the neuronal expression of SIFaR is critical for regulating both behaviors, regardless of their dependency on SIFa.

Knockdown of SIFaR in neurons marked by *fru-GAL4* or *dsx-GAL4* (which target overlapping populations) significantly reduced male CI (Figure 2C) but did not affect CL (Figure 2D). Given the known overlap between *fru*- and *dsx*-expressing neurons,^32^^,^^33^^,^^34^ these results indicate that CI requires SIFaR expression in the shared or distinct neuronal populations defined by these markers, while CL operates independently of SIFaR in these cell groups. In previous studies, we demonstrated that the *SIFaR*^*24F06*^*-GAL4* driver labels distinct neuronal populations in the SOG (subesophageal ganglion), OL (optic lobe), and AG (abdominal ganglion) regions, which overlap with those labeled by the *SIFaR*^*2A*^*-GAL4* line (generated using the chemoconnectome (CCT) knock-in method).^35^ Additionally, we found that SIFaR^24F06^ neurons are negative for both *fru* and *dsx* expression.^17^ Blocking synaptic transmission through TNT expression in either *fru*-negative SIFaR^24F06^ neurons, all SIFaR neurons, or specifically within *fru*-positive SIFaR neurons severely reduces CI and leads to an absence of successful mating attempts by males (Figures 2E and 2F). Thus, although CI and CL are differentially modulated by the expression of SIFaR in *fru*-positive neurons, both behaviors may necessitate the proper functioning of *fru*-positive SIFaR neurons.

Notably, inhibiting synaptic transmission in *fru*-positive SIFaR neurons does not affect the climbing ability of male flies (Figure S2A), suggesting that the decreased courtship activity or failure to mate is not attributable to locomotor impairments. It has also been reported that inhibiting *fruitless* neurons impacts courtship behavior without influencing locomotion.^36^ Considering that genetic controls exhibit normal sexual behavior (Figures S2B and S2C), these results imply that SIFaR-positive neurons are essential for generating both CI and CL (Figures 2C–2F). Furthermore, while SIFaR function is necessary for CI in both *fru*-positive and -negative neurons, it is only required in *fru*-negative neurons for CL generation (Figures 2E–2G).

Male’s mating duration (MD) in *Drosophila melanogaster* is a critical aspect of reproductive fitness, influenced by evolutionary pressures that shape mating strategies and behaviors. The MD in male fruit flies reflects adaptive responses to varying levels of competition and environmental contexts. Males exposed to high competition tend to exhibit longer mating durations, which can enhance their reproductive success by increasing the likelihood of successful fertilization, as we reported previously.^22^^,^^37^ This plasticity in mating duration allows males to adjust their reproductive investment based on social dynamics, optimizing their fitness in different mating scenarios. Our previous research^10^^,^^38^^,^^39^^,^^40^ and that of others^41^^,^^42^ has established various frameworks for investigating MD through advanced genetic techniques, allowing us to analyze the neural circuits that govern interval timing. Notably, males exhibit longer mating durations (LMD behavior) when faced with rival males, indicating an adaptive strategy to enhance reproductive success.^38^^,^^39^^,^^43^^,^^44^^,^^45^^,^^46^ Conversely, under sustained female-rich conditions (e.g., 1:2 male-to-female ratio), males exhibit Shorter Mating Duration (SMD) behavior—reducing copulation time to conserve reproductive effort for additional mating opportunities^47^^,^^48^ (See the quantification and interpretation of mating duration behaviors in STAR Methods section).

**Figure 3:**
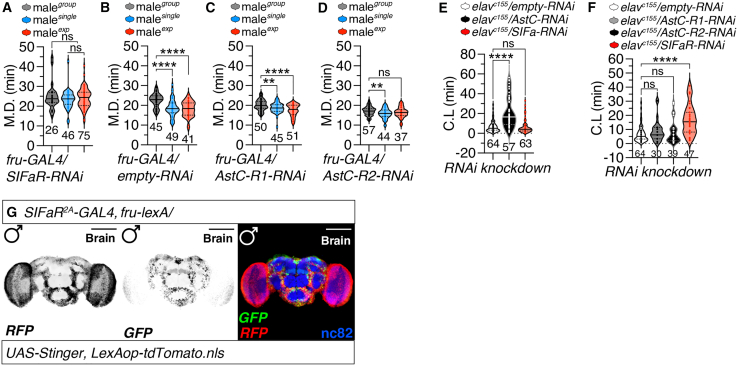
SIFaR and AstC signaling in fru-positive neurons regulate mating duration (A) Mating Duration (MD) assays of flies expressing the *fru-GAL4* driver together with *SIFaR-RNAi*. light gray violin plot denotes males that were group-reared (or sexually naive), whereas blue violin plot signifies males that were singly reared, and pink violin plot signify males that were sexual experienced. The dot plots represent the MD of each male fly. The mean value and standard error are labeled within the dot plot (black lines). Asterisks represent significant differences. For detailed methods, see the STAR Methods for a detailed description of the mating duration assays used in this study. Statistical significance determined by one-way ANOVA followed by Tukey’s multiple comparisons test. *p* = 0.5881 (one-way ANOVA, F = 0.53, R^2^ = 0.0073; Tukey’s post hoc). The ns represents non-significant differences. Sample sizes (n) are indicated in the figure panels. In the framework of our investigation, the routine application of internal controls is employed for the vast majority of experimental procedures, as delineated in the “mating duration assays” and “statistical tests” subsections of the STAR Methods section. The identical analytical approach employed for the MD assays is maintained for the subsequent data presented. Data are represented as mean ± SEM. (B) MD assays of flies expressing the *fru-GAL4* driver together with *empty-RNAi*. Statistical significance determined by one-way ANOVA followed by Tukey’s multiple comparisons test. *p* < 0.0001 (one-way ANOVA, F = 16.42, R^2^ = 0.20; Tukey’s post hoc). Sample sizes (n) are indicated in the figure panels. Data are represented as mean ± SEM. (C) MD assays of flies expressing the *fru-GAL4* driver together with *AstC-R1-RNAi.* Statistical significance determined by one-way ANOVA followed by Tukey’s multiple comparisons test. *p* < 0.0001 (one-way ANOVA, F = 10.72, R^2^ = 0.13; Tukey’s post hoc). Sample sizes (n) are indicated in the figure panels. Data are represented as mean ± SEM. (D) MD assays of flies expressing the *fru-GAL4* driver together with *AstC-R2-RNAi.* Statistical significance determined by one-way ANOVA followed by Tukey’s multiple comparisons test. *p* = 0.0286 (one-way ANOVA, F = 3.65, R^2^ = 0.05; Tukey’s post hoc). The ns represents non-significant differences. Sample sizes (n) are indicated in the figure panels. Data are represented as mean ± SEM. (E) CL of group-housed males expressing *elav*^*c155*^ together with *empty-RNAi, AstC-RNAi* and *SIFa-RNAi.* Statistical significance determined by one-way ANOVA followed by Tukey’s multiple comparisons test. *p* < 0.0001 (one-way ANOVA, F = 38.92, R^2^ = 0.30; Tukey’s post hoc). The ns represents non-significant differences. Sample sizes (n) are indicated in the figure panels. Data are represented as mean ± SEM. (F) CL of group-housed males expressing *elav*^*c155*^ together with *empty-RNAi, AstC-R1-RNAi, AstC-R2-RNAi* and *SIFaR-RNAi.* Statistical significance determined by one-way ANOVA followed by Tukey’s multiple comparisons test. *p* < 0.0001 (one-way ANOVA, F = 20.99, R^2^ = 0.28; Tukey’s post hoc). The ns represents non-significant differences. Sample sizes (n) are indicated in the figure panels. Data are represented as mean ± SEM. (G) Male flies expressing *GAL4*^*SIFa.2A*^ and *fru-lexA* together with *UAS-Stinger* and *LexAop-tdTomato.nls* were immunostained with anti-GFP (green), anti-RFP (red), nc82 (blue) antibodies. Scale bars represent 100 μm. For detailed methods, see the STAR Methods for a detailed description of the immunostaining procedure used in this study.

Our recent findings indicate that the long-range neuropeptide relay from SIFa to SIFaR plays a critical role in mediating LMD and SMD behaviors.^17^ To further explore whether SIFaR expression in *fru*-positive neurons contributes to LMD/SMD behaviors, we utilized RNAi to decrease SIFaR levels. The results showed that reducing SIFaR levels specifically within *fru*-positive neurons completely disrupted LMD/SMD behaviors when compared to genetic controls (Figures 3A and 3B). Recent single-cell RNA sequencing data from a fly cell atlas have identified several neuropeptide receptors (NPRs) that are highly enriched in *fru*- and *dsx*-positive neurons, suggesting their involvement in sexually dimorphic behaviors.^49^^,^^50^ Among these NPRs, AstC-R1 is particularly enriched in *fru*-positive neurons and has been associated with regulating insulin-producing cells (IPCs) that influence female egg-laying rhythms.^51^ Both AstC-R1 and AstC-R2 serve as receptors for the neuropeptide AstC; notably, AstC-R2 has been identified as a modulator of evening locomotor activity^52^ and is involved in food intake and metabolic homeostasis during nutrient stress in the gut.^53^

Despite both AstC-R1 and AstC-R2 being highly expressed in *fru*-positive neurons compared to SIFaR levels (Supporting Information 1), knocking down AstC-R1 did not alter LMD/SMD behaviors (Figure 3C), while knocking down AstC-R2 specifically affected SMD behavior (Figure 3D). These data indicate that the expression of AstC-R1 in *fru*-positive neurons is not necessary for LMD/SMD behaviors. Additionally, neuronal knockdown of AstC significantly delayed CL compared to controls or flies with reduced SIFa levels (Figure 3E), indicating that AstC release from these neurons contributes to CL phenotype. Interestingly, knockdown of both AstC-R1 and AstC-R2 did not impact CL phenotype relative to control or SIFaR knockdown flies, which exhibited significant delays in CL (Figure 3F), suggesting that signaling from AstC to its receptors for CL may involve non-neuronal pathways.

Previous studies have indicated that the functions of AstC-R1/R2 may extend into the gut for behavioral modulation.^53^^,^^54^ Furthermore, we found that many neurons expressing SIFaR also co-express FRU within the male brain (Figure 3G), reinforcing the notion that NPR expression levels do not necessarily correlate with specific fru-mediated functions (Supporting information 1). Overall, considering genetic controls for mating duration, these findings imply that the function of SIFaR within *fru*-positive neurons regarding sex-specific behaviors is distinctively unique (Figures S3A–S3D).

### Fru-specific SIFamide receptor expression in P1 and mAL neuronal populations specifically modulates mating duration, not copulation latency

**Figure 4:**
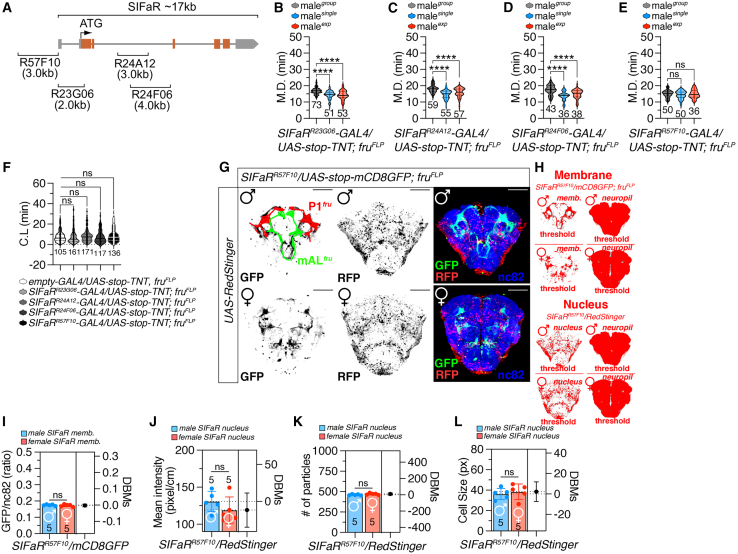
Specific SIFaR-expressing neurons regulate mating duration (A) Diagram of the captured enhancer region within each *SIFaR-GAL4* construct. (B) MD assays of flies expressing the *SIFaR*^*R23G06*^*-GAL4* driver together with *UAS-stop-TNT; fru*^*FLP*^*.* Statistical significance determined by one-way ANOVA followed by Tukey’s multiple comparisons test. *p* < 0.0001 (one-way ANOVA, F = 23.86, R^2^ = 0.22; Tukey’s post hoc). Sample sizes (n) are indicated in the figure panels. Data are represented as mean ± SEM. (C) MD assays of flies expressing the *SIFaR*^*R24A12*^*-GAL4* driver together with *UAS-stop-TNT; fru*^*FLP*^*.* Statistical significance determined by one-way ANOVA followed by Tukey’s multiple comparisons test. *p* = 0.5266 (one-way ANOVA, F = 0.6439, R^2^ = 0.0079; Tukey’s post hoc). Sample sizes (n) are indicated in the figure panels. Data are represented as mean ± SEM. (D) MD assays of flies expressing the *SIFaR*^*R24F06*^*-GAL4* driver together with *UAS-stop-TNT; fru*^*FLP*^*.* Statistical significance determined by one-way ANOVA followed by Tukey’s multiple comparisons test. *p* < 0.0001 (one-way ANOVA, F = 18.41, R^2^ = 0.24; Tukey’s post hoc). Sample sizes (n) are indicated in the figure panels. Data are represented as mean ± SEM. (E) MD assays of flies expressing the *SIFaR*^*R57F10*^*-GAL4* driver together with *UAS-stop-TNT; fru*^*FLP*^. Statistical significance determined by one-way ANOVA followed by Tukey’s multiple comparisons test. *p* = 0.3654 (one-way ANOVA, F = 1.01, R^2^ = 0.015; Tukey’s post hoc). The ns represents non-significant differences. Sample sizes (n) are indicated in the figure panels. Data are represented as mean ± SEM. (F) CL of group-housed males expressing *empty-GAL4* together with *UAS-stop-TNT; fru*^*FLP*^ and males expressing *SIFaR*^*R23G06*^*-GAL4, SIFaR*^*R24A12*^*-GAL4, SIFaR*^*R24F06*^*-GAL4* and *SIFaR*^*R57F10*^*-GAL4* driver together with *UAS-stop-TNT; fru*^*FLP*^. Statistical significance determined by one-way ANOVA followed by Tukey’s multiple comparisons test. *p* = 0.0778 (one-way ANOVA, F = 2.11, R^2^ = 0.012; Tukey’s post hoc). The ns represents non-significant differences. Sample sizes (n) are indicated in the figure panels. Data are represented as mean ± SEM. (G) Male(upper) and female(below) flies expressing *SIFaR*^*R57F10*^ and *fru*^*FLP*^ together with *UAS-RedStinger* and *UAS-stop-mCD8GFP* were immunostained with anti-GFP (green), anti-RFP (red), nc82 (blue) antibodies. The left two panels are presented as a gray scale to clearly show the GFP and RFP signal. Scale bars represent 50 μm. (H–L) Quantification of GFP fluorescence (H and I) and RFP fluorescence (E and J–L) in the male and female fly CB expressing *SIFaR*^*R57F10*^ and *fru*^*FLP*^ together with *UAS-RedStinger* and *UAS-stop-mCD8GFP*. (I) Quantification of GFP fluorescence in male and female CB. Bars represent the mean GFP fluorescence level with error bars representing SEM. Asterisks represent significant differences, as revealed by the Student’s t test and ns represents non-significant difference (*∗p < 0.05, ∗∗p < 0.01, ∗∗∗p < 0.001*). The same symbols for statistical significance are used in all other Figures. See the STAR Methods for a detailed description of the colocalization analysis used in this study. (J) Quantification of relative value for RFP intensity between male and female flies. See the STAR Methods for a detailed description of the fluorescence intensity analysis used in this study. (K–L) Quantification of particle number (K) and cell size (L) between male and female flies. See the STAR Methods for a detailed description of the particle analysis used in this study. Sample sizes (n) are indicated in the figure panels. Data are represented as mean ± SEM.

To identify the minimal region necessary for labeling GAL4 drivers that assess the functionality of SIFaR-mediated mating duration behaviors, we examined four custom GAL4 drivers targeting the SIFaR enhancer region (Figure 4A).^55^ Our previous work with these modified drivers demonstrated that the co-expression of *SIFaR-RNAi* with either *SIFaR*^*57F10*^*-GAL4* or *SIFaR*^*24F06*^*-GAL4* led to a complete loss of LMD and SMD behaviors, highlighting the essential role of SIFaR expression in these GAL4 driver-expressing cells for mating duration behaviors.^17^

Among these drivers, only inhibiting SIFaR^57F10^-positive and *fru*-positive neurons through TNT expression abolished both LMD and SMD behaviors compared to genetic controls (Figures 4B–4E; Figures S4A–S4E), suggesting that these specific neurons are crucial for modulating LMD/SMD behaviors. Notably, CL was unaffected by either the inhibition of SIFaR^57F10^- and *fru*-positive neurons (Figure 4F) or by knocking down SIFaR in these neuronal populations (Figure S4F), indicating their lack of involvement in CL regulation.

Visualization of SIFaR^57F10^-positive and *fru*-positive neurons revealed the presence of fruP1 and mAL neurons in the male central brain (CB) (top panels in Figure 4G; Figure S4G). Previous studies have established that fruP1-expressing neurons are vital for complex male courtship displays,^32^^,^^56^ while also directing female reproductive behaviors.^57^ In females, the neuronal populations labeled by SIFaR^57F10^ closely resemble those expressing fruP1 and mAL neurons (bottom panels in Figure 4G; Figure S4G). The membrane arborization coverage, fluorescence intensity, number of cells, and cell size of fru-positive neurons labeled by SIFaR^57F10^ are similar between males and females (Figures 4H–4L), suggesting shared neural circuits of *fru*-positive SIFaR neurons encoding sexually dimorphic behaviors in *Drosophila*.

### SIFamide receptor expression in GABAergic fru-positive mAL neurons regulates mating duration and courtship index but not copulation latency

**Figure 5:**
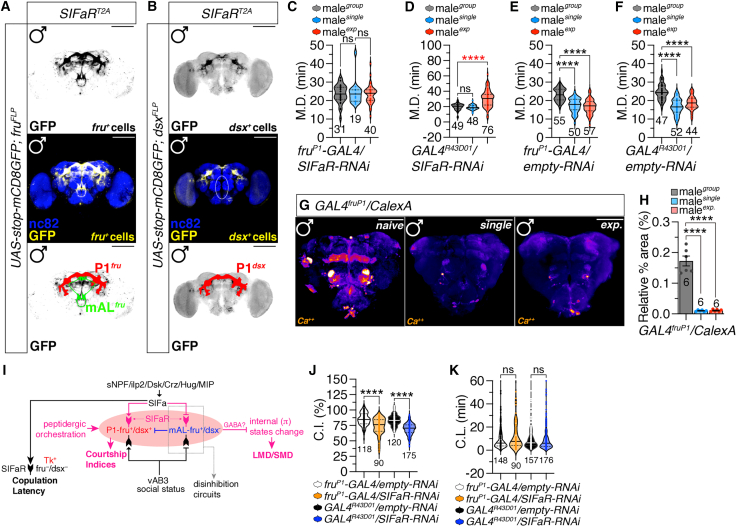
Fru-specific SIFaR expression in P1 and mAL neurons modulates mating duration (A) Male flies expressing *SIFaR*^*T2A*^ and *fru*^*FLP*^ together with *UAS-stop-mCD8GFP* were immunostained with anti-GFP (yellow), nc82 (blue) antibodies. (B) Male flies expressing *SIFaR*^*T2A*^ and *dsx*^*FLP*^ together with *UAS-stop-mCD8GFP* were immunostained with anti-GFP (yellow), nc82 (blue) antibodies. (C) MD assays of flies expressing the *fruP1-GAL4* driver together with *SIFaR-RNAi*. Statistical significance determined by one-way ANOVA followed by Tukey’s multiple comparisons test. *p* = 0.9192 (one-way ANOVA, F = 0.084, R^2^ = 0.0019; Tukey’s post hoc). The ns represents non-significant differences. Sample sizes (n) are indicated in the figure panels. Data are represented as mean ± SEM. (D) MD assays of flies expressing the *GAL4*^*R43D01*^ driver together with *SIFaR-RNAi*. Statistical significance determined by one-way ANOVA followed by Tukey’s multiple comparisons test. *p* < 0.0001 (one-way ANOVA, F = 44.07, R^2^ = 0.34; Tukey’s post hoc). The ns represents non-significant differences. Sample sizes (n) are indicated in the figure panels. Data are represented as mean ± SEM. (E) MD assays of flies expressing the *fruP1-GAL4* driver together with *empty-RNAi*. Statistical significance determined by one-way ANOVA followed by Tukey’s multiple comparisons test. *p* < 0.0001 (one-way ANOVA, F = 49.56, R^2^ = 0.38; Tukey’s post hoc). Sample sizes (n) are indicated in the figure panels. Data are represented as mean ± SEM. (F) MD assays of flies expressing the *GAL4*^*R43D01*^ driver together with *empty -RNAi*. Statistical significance determined by one-way ANOVA followed by Tukey’s multiple comparisons test. *p* < 0.0001 (one-way ANOVA, F = 47.72, R^2^ = 0.41; Tukey’s post hoc). The ns represents non-significant differences. Sample sizes (n) are indicated in the figure panels. Data are represented as mean ± SEM. (G) Different levels of neural activity of the brain as revealed by the CaLexA system in naive, single and experienced flies. Male flies expressing *fruP1*-*GAL4* along with *LexAop-CD2-GFP, UAS-mLexA-VP16-NFAT and LexAop-CD8-GFP-A2-CD8-GFP* were dissected after 5 days of growth (mated male flies had 1-day of sexual experience with virgin females). The dissected brains were then immunostained with anti-GFP (green) and anti-nc82 (blue). GFP is pseudo-colored as “red hot.” Scale bars represent 50 μm. (H) Quantification of relative intensity value for GFP fluorescence. Statistical significance determined by one-way ANOVA followed by Tukey’s multiple comparisons test. *p* < 0.0001 (one-way ANOVA, F = 97.94, R^2^ = 0.93; Tukey’s post hoc). The ns represents non-significant differences. Sample sizes (n) are indicated in the figure panels. Data are represented as mean ± SEM. (I) Diagram of interconnection between SIFaR and fru/dsx neurons along with interconnected networks formed by GAL4^24F06^. (J) CI of group-housed males expressing *fruP1-GAL4* together with *empty-RNAi* or *SIFaR-RNAi* and males expressing *GAL4*^*R43D01*^ with *empty-RNAi* or *SIFaR-RNAi*. Statistical significance determined by one-way ANOVA followed by Tukey’s multiple comparisons test. *p* < 0.0001 (one-way ANOVA, F = 70.43, R^2^ = 0.30; Tukey’s post hoc). The ns represents non-significant differences. Sample sizes (n) are indicated in the figure panels. Data are represented as mean ± SEM. (K) CL of group-housed males expressing *fruP1-GAL4* together with *empty-RNAi* or *SIFaR-RNAi* and males expressing *GAL4*^*R43D01*^ with *empty-RNAi* or *SIFaR-RNAi*. Statistical significance determined by one-way ANOVA followed by Tukey’s multiple comparisons test. *p* = 0.2605 (one-way ANOVA, F = 1.34, R^2^ = 0.007; Tukey’s post hoc). The ns represents non-significant differences. Sample sizes (n) are indicated in the figure panels. Data are represented as mean ± SEM.

Since the SIFaR promoter region drives SIFaR expression in the fruP1 and mAL regions, we systematically confirmed the fru-positive expression of SIFaR neurons utilizing a gene-specific T2A-GAL4 construct, *SIFaR*^*T2A*^*-GAL4*.^35^^,^^58^^,^^59^ As anticipated, the *SIFaR*^*T2A*^*-GAL4* driver labeled *fru*-positive P1 and mAL neurons in the male brain (Figure 5A). Notably, we observed that while the *dsx*-positive *SIFaR*^*T2A*^*-GAL4* driver marked P1 neurons, it did not label mAL neurons in the male brain (Figure 5B). This data indicates that SIFaR is expressed in *fru*-positive and *dsx*-positive P1 neurons but is restricted to *fru*-positive and *dsx*-negative mAL neurons.

We further validated that the expression patterns of the fruP1 and mAL-specific drivers exclusively colocalize with the *fru*-positive SIFaR^T2A^ driver, while showing no overlap with the *fru*-negative SIFaR^24F06^ driver (Figures S5A, S5B, and S5F). These findings align with our previous orthogonal verification using anti-FruM antibodies and nuclear markers as well as *fru*^*FLP*^ intersectional methods, confirming SIFaR^24F06^ neurons lack FruM protein expression.^17^ Given that mAL neurons are GABAergic and therefore exhibit characteristically low intracellular calcium concentrations under basal conditions, their Ca^2+^ levels fall below the minimum detection threshold of the CaLexA imaging system (Figure S5G). Consequently, these drivers enable us to explore the specific functions of SIFaR within both *fru*-positive and *fru*-negative interneuronal circuits. By utilizing these specific drivers, we can elucidate how SIFaR influences neural circuitry related to mating investment in a sexually dimorphic context.

Knockdown of SIFaR using fruP1- and mAL-specific *GAL4* drivers resulted in a complete disruption of both LMD/SMD behaviors compared to genetic controls (Figures 5C–5F). To determine whether ventral nerve cord (VNC) neurons modulate sexual behavior, we combined *tsh-GAL80* with *SIFaR* knockdown in fruP1 neurons. Notably, this manipulation did not alter key behavioral parameters—including mating duration, courtship index, or copulation latency—suggesting that VNC neurons are not critically involved in regulating these aspects of sexual behavior (Figures S5C–S5E). Notably, when SIFaR levels were specifically reduced in mAL neurons, MD in sexually experienced males was significantly longer compared to that of naive males (Figure 5D). The mAL neurons are GABAergic interneurons involved in courtship regulation. Recent studies have demonstrated that mAL neurons are activated by both male and female pheromones, underscoring their involvement in social interactions and behavioral responses to environmental stimuli. Based on their anatomical structure and neurotransmitter profile, mAL neurons have been proposed as sexually dimorphic GABAergic interneurons that convey inhibitory courtship signals to higher brain centers in males.^60^

Activation of the mAL cluster inhibits male courtship behavior toward females.^61^^,^^62^^,^^63^ At the circuit level, this regulation is mediated through GABA release from the mAL cluster into the main courtship command center of fruP1 neurons within the fly brain.^64^ This circuit receives inputs from interactions with females, which subsequently activate male-specific interneurons that trigger stereotypical courtship behavior in *Drosophila*.^65^ Our previous findings suggest that taste and pheromonal inputs via male-specific Gr5a/ppk25/ppk29 neurons into the brain are critical for generating SMD behavior.^48^ Thus, we hypothesize that the reversed SMD behavioral phenotype resulting from SIFaR knockdown in mAL neurons arises from inhibited GABAergic function within these neurons, leading to disinhibition (Figure 5I).

Interestingly, the knockdown of SIFaR in either fruP1 or mAL neurons resulted in a reduction of the CI (Figure 5J), without producing the increased courtship activity as shown in SMD behavior (Figure 5D). This finding suggests that MD may rely on disinhibitory circuits for male decision-making, whereas CI does not depend on the same disinhibitory pathways for context-dependent regulation (Figure 5J). Additionally, when monitoring fruP1 calcium dynamics using CalexA system, we observed that Ca^2+^ levels were significantly lower in single reared and sexually experienced males compared to naive controls (Figures 5G and 5H), further supporting the role of activity-dependent plasticity in these circuits. Knockdown of SIFaR in both fruP1 and mAL neurons did not affect CL (Figure 5K), indicating that CL is likely modulated by *fru*-negative SIFaR-expressing neurons. Collectively, these results imply that various mating behaviors in males can be regulated by the same SIFa-to-SIFaR circuitry through different regional expressions of SIFaR and distinct circuit combinations (Figure 5I).

To identify the specific neuronal populations responsible for modulating CL without affecting CI or MD, we conducted a mini screen of previously reported neuropeptidergic circuits. We discovered that knocking down SIFaR in neuropeptide tachykinin (Tk)-positive neurons specifically delayed CL without impacting LMD/SMD behaviors (Figures S5H and S5I). Tk neurons have been shown to promote aggression in male *Drosophila*.^66^ When Tk neuropeptide was knocked down in SIFaR-positive neurons, LMD/SMD behaviors remained intact, but CL was significantly delayed (Figures S5J and S5K), suggesting that the SIFa-to-SIFaR/Tk pathway specifically modulates CL (Figure 5I). These findings highlight the nuanced roles of different neuropeptidergic circuits in regulating distinct aspects of mating behavior. By elucidating these pathways, we can gain deeper insights into how neuropeptidergic signaling influences mating investment and behavioral outcomes in *Drosophila*.

### The synaptic alterations observed in mAL neurons are associated with distinct social contexts

**Figure 6:**
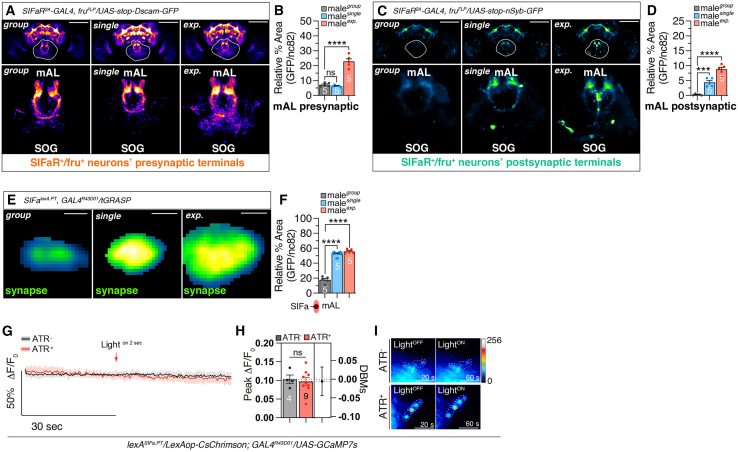
Synaptic alterations in mAL neurons correlate with social contexts (A) The SIFaR^+^/fru^+^ presynaptic terminals visualized by males expressing *SIFaR*^*2A*^*-GAL4, fru*^*FLP*^ together with *UAS-stop-Dscam-GFP* in naive, single and exp. male flies. Scale bars represent 100 μm. (B) Quantification of relative value for presynaptic terminals intensity. Statistical significance determined by one-way ANOVA followed by Tukey’s multiple comparisons test. *p* < 0.0001 (one-way ANOVA, F = 70.55, R^2^ = 0.92; Tukey’s post hoc). The ns represents non-significant differences. Sample sizes (n) are indicated in the figure panels. Data are represented as mean ± SEM. (C) The SIFaR^+^/fru^+^ postsynaptic terminals visualized by males expressing *SIFaR*^*2A*^*-GAL4, fru*^*FLP*^ together with *UAS-stop-nSyb-GFP* in naive, single and exp. male flies. Scale bars represent 100 μm. (D) Quantification of relative value for postsynaptic terminals intensity. Statistical significance determined by one-way ANOVA followed by Tukey’s multiple comparisons test. *p* < 0.0001 (one-way ANOVA, F = 59.83, R^2^ = 0.91; Tukey’s post hoc). The ns represents non-significant differences. Sample sizes (n) are indicated in the figure panels. Data are represented as mean ± SEM. (E) The synaptic interactions visualized utilizing the tGRASP system in naive, single and exp. male flies. (F) Quantification of synaptic puncta formed between *SIFa*^*lexA.PT*^ and *GAL4*^*R43D01*^ in brain between naive, single and exp. male flies. Statistical significance determined by one-way ANOVA followed by Tukey’s multiple comparisons test. *p* < 0.0001 (one-way ANOVA, F = 202.1, R^2^ = 0.96; Tukey’s post hoc). The ns represents non-significant differences. Sample sizes (n) are indicated in the figure panels. Data are represented as mean ± SEM. (G–I) mAL neurons don’t respond to SIFas activation. Fluorescence changes (Δ*F*/*F*_0_) of GCaMP7s in mALs after the optogenetic stimulation of SIFas (two-tailed unpaired *t*-test). In all plots and statistical tests. Data are presented as mean ± s.e.m. ns = not significant (*p > 0.05*), *∗p < 0.05, ∗∗p < 0.01, ∗∗∗p < 0.001, ∗∗∗∗p < 0.0001*. DBMs represent the ‘difference between means’ for the evaluation of estimation statistics (See STAR Methods). Sample sizes (n) are indicated in the figure panels. Data are represented as mean ± SEM. LED light was fired 2 s after 30 s of dark. Red arrows indicate the time point of light stimulation. *N* = 4 to 9 in each group. Red lines and bars indicate experimental group fed with ATR; black lines and bars indicate control group fed without ATR. See the STAR Methods for a detailed description of the two-photon calcium imaging used in this study.

To understand how mAL neurons differentially regulate LMD and SMD behaviors, we conducted a genetic intersection analysis to visualize the presynaptic and postsynaptic terminals of *fru*-positive and *SIFaR*-positive neurons.^67^ Notably, the presynaptic terminals of *SIFaR*^*+*^*/fru*^*+*^ mAL neurons exhibited a significant increase in sexually experienced males (Figures 6A and 6B). In contrast, the postsynaptic terminals of mAL neurons were enhanced in both socially isolated and sexually experienced males (Figures 6C and 6D). We also found that both social isolation and sexual encounters precipitated an augmentation in the synaptic connectivity between these neuronal populations (Figures 6E and 6F). The intensified DsCam-GFP, nSyb-GFP, and tGRASP signals in experienced males (Figures 6A–6F) may arise in part from elevated SIFaR expression induced by social and sexual experience, consistent with our prior transcriptomic data.^18^^,^^68^ Thus, while indicative of enhanced connectivity, these results cannot exclude contributions from expression-level changes of the receptor.

We identified that only one cell in each hemisphere of the mAL neurons expresses SIFaR (Figure S6A). To investigate the synaptic architecture linking SIFa and mAL neurons within the brain under diverse social conditions, we observed that the signal was strictly restricted in the SIFaR^+^/mAL^+^ cells as Figure S6A demonstrated. To validate the specificity of our newly generated *lexA*^*SIFa.PT*^ driver, we compared its expression pattern with that of *GAL4*^*SIFa.PT*^ is using a membrane marker in the fly brain and VNC. Although *lexA*^*SIFa.PT*^ exhibited weaker expression in some regions; the four *SIFa*-expressing cells showed complete overlap between the two drivers (Figure S6B). This precise colocalization confirms the successful generation of *lexA*^*SIFa.PT*^ and supports the reliability of our experimental results.

To investigate whether mAL neurons respond to SIFa neuron activation, we performed live calcium (Ca^2+^) imaging in mAL neurons using two-photon microscopy. A genetically encoded calcium indicator, GCaMP7s, was expressed in mAL neurons under the control of the *lexA*^*SIFa.PT*^ driver. To selectively activate SIFa neurons, we employed an optogenetic approach using the red-light-sensitive channelrhodopsin CsChrimson, expressed via *lexA*^*SIFa.PT*^ (Figure 6G). Upon the optogenetic stimulation of SIFa neurons, we observed no significant change in Ca^2+^ activity in mAL neurons, which remained stable and comparable to control flies lacking all-trans retinal (ATR) (Figures 6H and 6I). These results indicate that mAL neurons do not respond to SIFa neuron activation, likely due to their GABAergic nature, which may inhibit downstream signaling. These findings suggest that SIFa inputs to this unique *fru*^*+*^*/SIFaR*^*+*^ mAL neuron create context-specific internal states by modulating synaptic plasticity within these neurons. The genotypes of all *Drosophila* used in each figure of this study are detailed in Table 1.

## Discussion

The results of our study reveal that SIFa neurons play a critical role in orchestrating male sexual behaviors, specifically CI, CL, and MD. We found that reducing SIFa peptide levels through RNA interference significantly decreased male CI toward females, indicating the importance of SIFa release for normal courtship behavior. In contrast, altering neuronal activity in SIFa-positive neurons did not affect CI, suggesting that the neuropeptide’s release is more crucial than the activity of SIFa-expressing neurons themselves. Regarding CL, we observed that inhibiting SIFa neuronal activity led to delays in copulation initiation, while the hyperactivation of these neurons had no effect. This indicates that the active states of SIFa neurons are essential for maintaining rapid CL, unlike CI, which is dependent on synaptic transmission involving dopamine. Our analysis of neuropeptide receptors indicated that sNPF signaling is vital for both CI and CL modulation, while insulin and hugin signaling specifically regulate CI, and Crz signaling influences CL.

We also established that SIFaR expression in *fru*-positive neurons is critical for regulating MD and CI but not CL. Knockdown of SIFaR in fruP1 and mAL neurons disrupted both LMD and SMD behaviors, highlighting their essential role in mating investment. Importantly, sexually experienced males exhibited longer MD when SIFaR levels were reduced specifically in mAL neurons compared to naive males. Overall, these findings underscore the distinct yet interconnected neural mechanisms underlying male sexual behavior, emphasizing the role of SIFa and its receptors in shaping reproductive strategies in *Drosophila melanogaster*. This research highlights the intricate relationship between sensory input and behavioral output in *Drosophila* mating strategies. The results of our study reveal that both social isolation and instances of sexual activity result in enhanced synaptic connectivity within the SIFa to mAL neuronal pathway (Figures 6E and 6F). This observation implies that synaptic plasticity is a pivotal mechanism in adapting the SIFa-mAL circuitry based on varying social environments. The modulation of synaptic plasticity in response to contextual cues emphasizes the adaptability of neural circuits involved in mating behaviors. Our findings contribute to a deeper understanding of how neuropeptidergic signaling can fine-tune behavioral responses based on social and sexual experiences. We further propose that SIFa-to-SIFaR signaling induces distinct internal brain states in CI and CL, potentially mediated by varying neuropeptidergic input thresholds to SIFa neurons,^69^^,^^70^^,^^71^ differential reliance on neurotransmitter systems, and downstream neuronal regulation of SIFaR-expressing neurons. However, additional experiments are required to validate this hypothesis and elucidate the precise mechanisms involved (Figure 1I).

Our results reveal a functional segregation within SIFa-positive neurons that CI depends exclusively on DA signaling, while CL requires Glu/OA/TA transmission. To explain the SIFa’s release independence and the apparent discrepancy in *ple* knockdown effects, we propose a mechanistic model. We hypothesize that SIFa, as a neuropeptide, is released via dense-core vesicles (DCVs) through calcium-dependent but SNARE-independent mechanisms,^72^^,^^73^ rendering it resistant to TNT or depolarization-blocking agents such as KCNJ2. This non-canonical release mechanism enables SIFa to regulate neuronal activity independent of conventional vesicular fusion machinery, while still maintaining the capacity to modulate CI through specific neural circuits.

In this dual-transmitter architecture, SIFa acts as a master regulator biasing co-released neurotransmitters toward distinct behavioral outputs. Regarding CI, SIFa potentiates DA signaling via autocrine feedback mechanisms, likely through the SIFa receptor (SIFaR)-mediated enhancement of tyrosine hydroxylase activity or dopamine vesicle loading. This is consistent with our previous demonstration of SIFaR expression in SIFa neurons themselves.^18^ This explains why *ple* knockdown (disrupting DA synthesis) impairs CI (Figure 1E) while CL remains unaffected (Figure 1F). For CL, SIFa independently potentiates Glu/OA/TA transmission through ionotropic receptor modulation. Regarding the query about TNT/KCNJ2 effects on DA release (Figure 1A), we posit that SIFa’s TNT-resistant release compensates by either: (i) directly activating postsynaptic DA receptors via volume transmission, or (ii) sustaining baseline DA tone through enhanced *ple*-mediated synthesis, even when exocytosis is impaired. Thus, while *ple* knockdown causes irreversible DA depletion, TNT/KCNJ2 may only transiently block exocytosis, leaving SIFa-mediated DA signaling partially functional.

This model predicts that SIFa knockout would abolish both CI and CL, whereas TNT/KCNJ2 expression in SIFa neurons would impair CL (via disrupted Glu/OA/TA release) but spare CI due to SIFa’s compensatory actions. Future studies should validate these predictions through intersectional manipulations of SIFa and specific neurotransmitter systems. Collectively, our findings suggest SIFa neurons employ hierarchical release strategies, with neuropeptide-mediated gating ensuring robust behavioral modulation independent of conventional release pathways.

The P1 (pC1) neurons in *Drosophila melanogaster* are a pivotal cluster of male-specific neurons that integrate sensory information and modulate behaviors related to mating and aggression. Recent studies have highlighted the role of neuropeptidergic inputs in regulating the activity of these neurons, thereby influencing male mating investment and behavioral outcomes. P1 neurons express a diverse array of neuropeptides that play critical roles in modulating their activity. For instance, recent transcriptional profiling revealed that these neurons are enriched in genes encoding neuropeptides such as Neuropeptide F (NPF), Drosulfakinin (Dsk), and others that are implicated in behavioral regulation.^49^^,^^74^ The activation of P1 neurons can lead to both aggressive and mating behaviors, suggesting that the balance of neuropeptidergic signaling is crucial for determining behavioral outcomes in different social contexts.^75^ Moreover, P1 neurons have been shown to be influenced by external factors such as food availability and social interactions. High-quality food and starvation conditions modulate P1 neuron activity through insulin and tyramine signaling pathways, indicating a sophisticated interplay between nutritional state and mating drive.^76^ This modulation underscores the context-dependent nature of neuropeptide signaling in shaping persistent internal states for mating investment.

SIFa neurons exhibit extensive branching throughout the central nervous system and integrate diverse inputs from multiple neuropeptide signals, playing a crucial role in regulating internal states related to hunger, satiety, and social status.^1^^,^^14^^,^^18^ Our study provides significant insights into how SIFa inputs to fruP1 central neurons can orchestrate various sexual behaviors in a context-dependent manner. Specifically, we found that the modulation of MD and CI is influenced by SIFa signaling, with distinct neural mechanisms governing these behaviors. The results indicate that while SIFaR expression in *fru*-positive neurons is essential for CI, it does not impact CL. Additionally, the differential responses of mAL neurons to social contexts further underscore the complexity of SIFa’s role in shaping male sexual behaviors. Overall, our findings shed light on the mechanistic details of prior research indicating that SIFa influences sexual behavior through its action on *fruitless* neurons via SIFaR.^15^^,^^16^

One intriguing finding is that CL is not influenced by SIFa release (Figure 1D) or NT release from SIFa neurons (Figure 1F), yet it is affected by the neuronal knockdown of SIFaR (Figure 2B). In fact, CL relies on specific neuropeptide inputs to SIFa neurons, particularly Crz and sNPF (Figure 1H). This raises the question of how CL can be modulated by SIFa inputs and neuronal SIFaR expression while remaining unaffected by SIFa expression or NT release from SIFa neurons. We propose that non-synaptic transmission from SIFa to *fru*-negative SIFaR neurons may account for this phenomenon. Neurons can indeed influence behavior independently of traditional neurotransmitter systems.^77^^,^^78^^,^^79^

Disinhibition, characterized by the alleviation of inhibitory constraints, allows for the activation of neural circuits typically suppressed. This mechanism is vital for shaping behavioral patterns and facilitating the sequential progression of actions. By promoting selective neuronal activation while concurrently inhibiting competing neural pathways, disinhibition enables the brain to dynamically assess and maintain necessary behavioral states while facilitating transitions to subsequent behavioral phases.^80^ It is also recognized that *Drosophila* neural circuits exhibit disinhibition phenotypes related to light preference and ethanol sensitization.^81^^,^^82^

Additionally, research has shown that certain behaviors can remain intact even when specific neurotransmitter systems are genetically disrupted. For example, studies on dopamine neurons have indicated that while glutamatergic inputs are important for certain effort-related tasks, other aspects of behavior may not be significantly affected by the absence of these inputs.^83^ This suggests that neurons can utilize various signaling modalities to influence behavior, including non-neurotransmitter signaling pathways. Moreover, the presence of ‘non-neurotransmitter signaling’ pathways, such as neuroinflammatory responses and other chemical mediators such as nitric oxide, further illustrates how neuronal and non-neuronal interactions contribute to behavioral outcomes.^84^ Thus, it is clear that while neurotransmitters play a vital role in neuronal communication and behavior, there are multiple mechanisms through which neurons can affect behavior independently of traditional neurotransmitter systems.^84^^,^^85^^,^^86^^,^^87^ The stable phenotype of CL may suggest the involvement of non-synaptic transmission mechanisms as regulatory controls.

It is well established that mAL neurons receive pheromonal inputs from both female and male sources through vAB3 neuronal circuits and inhibit fruP1 neurons via GABA release.^62^ Integrating these observations with our data, we propose that the differential pheromonal inputs from males and females may be orchestrated by specific SIFaR expression in targeted P1 and mAL neurons to elicit distinct behavioral responses (Figure S6C).^88^

While our RNAi experiments implicate the genes we tested in regulating LMD/SMD and CI/CL behaviors, we note that RNAi-mediated knockdown may not achieve complete loss-of-function. The lines used in our study were pre-validated in both prior studies^12^^,^^26^^,^^27^^,^^28^^,^^51^^,^^53^^,^^89^^,^^90^^,^^91^^,^^92^^,^^93^^,^^94^^,^^95^^,^^96^^,^^97^ and our lab controls (*UAS-RNAi*/+, *GAL4*/+), showing consistent phenotypic effects. We interpret reduced effects as “attenuation” rather than abolition unless supported by null mutants or rescue experiments. Future studies using CRISPR or tissue-specific rescue could further validate these findings, but our results remain robust within RNAi limitations.

In conclusion, our study elucidates the critical role of SIFa neurons in orchestrating male sexual behaviors through distinct neuropeptidergic signaling pathways. By demonstrating how SIFa influences various sexual behaviors via different mechanisms, we provide valuable insights into the complex interplay between neuropeptides and sexual behavior, underscoring the importance of context-dependent regulation in *Drosophila melanogaster*.

### Limitations of the study

While our study provides insights into the role of SIFa/SIFaR signaling in male mating behaviors, several limitations should be acknowledged. First, SIFa might influence copulation latency through non-synaptic volume transmission, but this remains hypothetical without direct evidence of neuropeptide diffusion dynamics *in vivo*. Cutting-edge tools such as genetically encoded neuropeptide sensors (e.g., GRAB reporters) combined with *in vivo* imaging could experimentally test this proposed mechanism. Second, while we standardized behavioral assays using SPR^−/−^ females, environmental variability might still influence results. Future studies could employ connectomics, or real-time neuropeptide imaging to address these gaps.

## Resource availability

### Lead contact

Requests for further information and resources should be directed to and will be fulfilled by the lead contact, Woo Jae Kim (wkim@hit.edu.cn).

### Materials availability

Strains are available upon request.

### Data and code availability

• All data reported in this article will be shared by the lead contact upon request.
• This article does not report original code. The URL of the codes used in this article are listed in the key resources table.
• Any additional information required to reanalyze the data in this article is available from the lead contact upon request.

## Acknowledgments

We thank Dr. Jan A. Veenstra (University of Bordeaux) for sharing *the SIFaPT-GAL4 driver, Dr. Young-Joon Kim (GIST) for sharing the UAS-stop-nSyb-GFP* and *UAS-stop-Dscam17.1-EGFP* fly strains. We gratefully acknowledge Dr. Yufeng Pan (Southeast University) for generously providing the fruP1 and mAL driver lines, as well as for sharing valuable insights into sexually dimorphic circuit analysis. We gratefully acknowledge Dr. Wei Zhang (Tsinghua University) for his valuable guidance and expert advice on our GCaMP live imaging experiments. We have greatly benefited from the resources provided by the FlyBase website in our genetic research endeavors. We are grateful for the ongoing efforts of the FlyBase staff in maintaining this comprehensive *Drosophila* database.^98^^,^^99^^,^^100^^,^^101^^,^^102^ This research was supported by Startup funds from HIT Center for Life Science to W.J.K. The funder had no role in study design, data collection and analysis, decision to publish, or preparation of the article.

## Author contributions

Conceptualization: W.J.K.; data curation: T.Z., H.M., Y.S., Z.W., and W.J.K.; formal analysis: T.Z., H.M., Y.S., Z.W., and W.J.K.; funding acquisition: W.J.K.; investigation: W.J.K.; methodology: W.J.K.; project administration: W.J.K.; resources: W.J.K.; supervision: W.J.K.; validation: T.Z., H.M., Y.S., and W.J.K.; visualization: Z.W. and W.J.K.; writing – original draft: W.J.K.; writing – review and editing: T.Z. and W.J.K.

## Declaration of interests

The authors declare no competing interests.

## Declaration of generative AI and AI-assisted technologies in the writing process

During the creation of this work, the author(s) utilized deepseek AI (https://chat.deepseek.com/) to rephrase English sentences, verify English grammar, and detect plagiarism, as none of the authors of this article are native English speakers. After using this tool/service, the author(s) reviewed and edited the content as needed and take(s) full responsibility for the content of the publication.

## STAR★Methods

### Key resources table

**Table:** 

REAGENT or RESOURCE	SOURCE	IDENTIFIER
**Antibodies**		

Chicken polyclonal anti-GFP	Invitrogen	RRID: AB_2534023
Donkey Alexa-488 anti-chicken	Jackson ImmunoResearch	RRID: AB_2340375
Goat Alexa-555 anti-rabbit	Invitrogen	RRID: AB_2535849
Goat Alexa-647 anti-mouse	Jackson ImmunoResearch	RRID: AB_2338902
Mouse polyclonal anti-nc82	Developmental Studies Hybridoma Bank	RRID: AB_2314866
Rabbit polyclonal anti-RFP	Rockland Immunochemicals	RRID: AB_2209751

**Bacterial and virus strains**		

pBPLexA::p65Uw	Gerald Rubin	Addgene 26231
E2 Enhancer-lexA	Qidong Fungene Biotechnology	N/A
DH5α Chemically Competent Cell	Weidibio	DL1001

**Chemicals, peptides, and recombinant proteins**		

Triton X-100	Alphabio	A2576
4% Polyformaldehyde	Alphabio	A1283
Mounting solution	Solarbio	S2100-5
PBS	Solarbio	P1022-500

**Critical commercial assays**		

FastDigest EcoRI	Thermo Scientific	FD0274
XbaI	NEB	R0145S
DNAzol BD	Invitrogen	10974020
PrimeSTAR HS DNA Polymerase	Takara	R010A
Light Cloning kit	Biodragon (China)	BDIT0014

**Experimental models: Organisms/strains**		

*D. melanogaster: Deficiency females:* *Df(1)*^*Exel6234*^	Bloomington Drosophila Stock Center	RRID: BDSC_7708
*D. melanogaster: genetic control:* *UAS-GFP.nls*	Bloomington Drosophila Stock Center	RRID: BDSC_4775
*D. melanogaster: pan neuronal driver:* *elav*^*c155*^*; UAS-Dicer*	Bloomington Drosophila Stock Center	RRID: BDSC_25750
*D. melanogaster: CalexA system:* *lexAop-CD8GFP; UAS-mLexA-VP16-NFAT, lexAop-rCD2-GFP*	Bloomington Drosophila Stock Center	RRID: BDSC_66542
*D. melanogaster: tGRASP system:* *UAS-post-t-GRASP, lexAop2-pre-t-GRASP*	Bloomington Drosophila Stock Center	RRID: BDSC_79039
*D. melanogaster:SIFa-RNAi:* *SIFa-RNAi*	Bloomington Drosophila Stock Center	RRID: BDSC_60484
*D. melanogaster: SIFa.PT-GAL4 driver:* *SIFa-GAL4*	Jan A Veenstra	N/A
*D. melanogaster: stop-GFP marker:* *UAS-FRT-stop-FRT-mCD8GFP*	Bloomington Drosophila Stock Center	RRID: BDSC_30125
*D. melanogaster: SIFa*.*PT-lexA driver: SIFa-lexA*	This paper	STAR Methods
*D. melanogaster: pan glial driver:* *repo-GAL4*	Bloomington Drosophila Stock Center	RRID: BDSC_7415
*D. melanogaster: control GAL4:* *empty-GAL4*	Bloomington Drosophila Stock Center	RRID: BDSC_68384
*D. melanogaster: control RNAi:* *empty-RNAi*	Bloomington Drosophila Stock Center	RRID: BDSC_36304
*D. melanogaster: SIFaR-23G06-GAL4 driver:* *GAL4*^*23G06*^	Bloomington Drosophila Stock Center	RRID: BDSC_49041
*D. melanogaster: SIFaR-24A12-GAL4 driver:* *GAL4*^*24A12*^	Bloomington Drosophila Stock Center	RRID: BDSC_49061
*D. melanogaster: SIFaR-24F06-GAL4 driver:* *GAL4*^*24F06*^	Bloomington Drosophila Stock Center	RRID: BDSC_49087
*D. melanogaster: SIFaR-57F10-GAL4 driver:* *GAL4*^*57F10*^	Bloomington Drosophila Stock Center	RRID: BDSC_46391
*D. melanogaster: SIFaR-T2A-GAL4 driver:* *SIFaR-GAL4*^*T2A*^	Qidong Fungene Biotechnology	RRID: FBF00102
*D. melanogaster: SIFaR-24F06-lexA driver:* *lexA*^*24F06*^	Bloomington Drosophila Stock Center	RRID: BDSC_52695
*D. melanogaster: SIFaR-T2A-lexA driver:* *SIFaR-lexA*^*T2A*^	Qidong Fungene Biotechnology	RRID: FBF00086
*D. melanogaster: Open rectifier K*^*+*^*channel 1:* *UAS-Ork1.Delta-C*	Bloomington Drosophila Stock Center	RRID: BDSC_6586
*D. melanogaster: ple-RNAi:* *ple-RNAi*	Bloomington Drosophila Stock Center	RRID: BDSC_25796
*D. melanogaster: NaChBac:* *UAS-NaChBac*	Bloomington Drosophila Stock Center	RRID: BDSC_9467
*D. melanogaster: ChaT-RNAi* *ChaT-RNAi*	Bloomington Drosophila Stock Center	RRID: BDSC_25856
*D. melanogaster: double sex driver:* *dsx-GAL4*	Bloomington Drosophila Stock Center	RRID: BDSC_30027
*D. melanogaster: Gad1-RNAi:* *Gad1-RNAi*	Bloomington Drosophila Stock Center	RRID: BDSC_51794
*D. melanogaster: nucleus fluorescent marker:* *UAS-RedStinger*	Bloomington Drosophila Stock Center	RRID: BDSC_8546
*D. melanogaster: Nucleus fluorescent marker:* *UAS-Stinger, lexAop-tdTomato.nls*	Bloomington Drosophila Stock Center	RRID: BDSC_66680
*D. melanogaster: CrzR-RNAi:* *CrzR-RNAi*^*JF02042*^	Bloomington Drosophila Stock Center	RRID: BDSC_26017
*D. melanogaster: VGlut-RNAi:* *VGlut-RNAi*	Bloomington Drosophila Stock Center	RRID: BDSC_27538
*D. melanogaster: Lgr4-RNAi:* *Lgr4-RNAi*	Bloomington Drosophila Stock Center	RRID: BDSC_28655
*D. melanogaster: potassium channel activator:* *UAS-KCNJ2*	Bloomington Drosophila Stock Center	RRID: BDSC_6596
*D. melanogaster: membrane marker:* *UAS-mCD8RFP, lexAop-mCD8GFP*	Bloomington Drosophila Stock Center	RRID: BDSC_32229
*D. melanogaster: chemical synaptic transmission blocker:* *UAS-TNT*	Bloomington Drosophila Stock Center	RRID: BDSC_28838
*D. melanogaster: Lgr3-RNAi:* *Lgr3-RNAi*	Bloomington Drosophila Stock Center	RRID: BDSC_28789
*D. melanogaster: CCKLR-17D3-RNAi:* *CCKLR-17D3-RNAi*	Bloomington Drosophila Stock Center	RRID: BDSC_28333
*D. melanogaster: CCKLR-17D1-RNAi:* *CCKLR-17D1-RNAi*	Bloomington Drosophila Stock Center	RRID: BDSC_27494
*D. melanogaster: sNPF-RNAi:* *sNPF-RNAi*	Bloomington Drosophila Stock Center	RRID: BDSC_27507
*D. melanogaster: fruitless driver:* *fru-GAL4*	Bloomington Drosophila Stock Center	RRID: BDSC_30027
*D. melanogaster: fruitless driver:* *fru-GAL4*	Bloomington Drosophila Stock Center	RRID: BDSC_30027
*D. melanogaster: fruitless driver:* *fru-GAL4*	Bloomington Drosophila Stock Center	RRID: BDSC_30027
*D. melanogaster: temperature sensitive tub-GAL80:* *tub-GAL80*^*ts*^	Bloomington Drosophila Stock Center	RRID: BDSC_7017
*D. melanogaster: fruP1 driver:* *fruP1-GAL4*	Bloomington Drosophila Stock Center	RRID: BDSC_66696
*D. melanogaster: fru-FLP:* *fru-FLP*	Bloomington Drosophila Stock Center	RRID: BDSC_66870
*D. melanogaster: UAS-stop-TNT:* *UAS-stop-TNT*	Bloomington Drosophila Stock Center	RRID: BDSC_28842
*D. melanogaster: UAS-stop-TNT inactive form:* *UAS-stop-TNT*^*in*^	Bloomington Drosophila Stock Center	RRID: BDSC_28844
*D. melanogaster: AstC-R2-RNAi:* *AstC-R2-RNAi*	Bloomington Drosophila Stock Center	RRID: BDSC_25940
*D. melanogaster: mAL driver:* *R43D01-GAL4*	Bloomington Drosophila Stock Center	RRID: BDSC_64345
*D. melanogaster: Tk driver:* *Tk-GAL4*	Bloomington Drosophila Stock Center	RRID: BDSC_93412
*D. melanogaster: Trhn-RNAi:* *Trhn-RNAi*	Bloomington Drosophila Stock Center	RRID: BDSC_25842
*D. melanogaster: Tdc2-RNAi:* *Tdc2-RNAi*	Bloomington Drosophila Stock Center	RRID: BDSC_25871
*D. melanogaster: Tk-RNAi:* *Tk-RNAi*	Bloomington Drosophila Stock Center	RRID: BDSC_25800
*D. melanogaster: CCHa1-R-RNAi:* *CCHa1-R-RNAi*	Vienna *Drosophila* Resource Center	RRID: VDRC_103055
*D. melanogaster: InR-RNAi:* *InR-RNAi*	Vienna *Drosophila* Resource Center	RRID: VDRC_991
*D. melanogaster: PK2-R1-RNAi:* *PK2-R1-RNAi*	Vienna *Drosophila* Resource Center	RRID: VDRC_103822
*D. melanogaster: PK2-R2-RNAi:* *PK2-R2-RNAi*	Vienna *Drosophila* Resource Center	RRID: VDRC_44871
*D. melanogaster: AstC-R1-RNAi:* *AstC-R1-RNAi*	Vienna *Drosophila* Resource Center	RRID: VDRC_13560
*D. melanogaster: nSyb GFP marker:* *UAS-stop-nSyb-GFP*	Young-Joon Kim	N/A
*D. melanogaster: Dscam GFP marker:* *UAS-stop-Dscam17.1-EGFP*	Young-Joon Kim	N/A

**Oligonucleotides**		

Primer: SIFa Forward: GCCAATTGGCTGAATCTCCTGACCCTCA	Jan A Veenstra	N/A
Primer: SIFa Reverse: GCAGATCTCTTGCAGTTTTCGGTGAGC	Jan A Veenstra	N/A

**Software and algorithms**		

Fiji/ImageJ	Schindelin et al., 2012	https://fiji.sc
Prism 9.0	GraphPad	https://www.graphpad.com
Adobe illustrator 2020	Adobe.com	N/A
SCOPE	Fly Cell Atlas	https://scope.aertslab.org/#/FlyCellAtlas/∗/welcome
Seurat	Yuhan et al., 2023	https://github.com/satijalab/seurat
R v4.2.2	R Core Team	https://www.R-project.org/
QuillBot AI	QuillBot Core Team	https://quillbot.com/
Microsoft Office Excel	Microsoft	https://www.microsoft.com/en-au/microsoft-365/excel

### Experimental model and study participant details

#### Drosophila

*Drosophila melanogaster* were cultured under standard laboratory conditions at 25°C. Samples were prepared as described in the method details. All fly strains are listed in the key resources table.

#### Statement of animal research compliance

All animal experiments reported in this article were conducted in compliance with the ARRIVE guidelines and adhered to the U.K. Animals (Scientific Procedures) Act, 1986 and associated guidelines, EU Directive 2010/63/EU for animal experiments, or the National Research Council’s Guide for the Care and Use of Laboratory Animals.

### Method details

#### Fly stocks and husbandry

*Drosophila melanogaster* were raised on cornmeal-yeast medium at similar densities to yield adults with similar body sizes. For 20 liters of fly food, the composition includes 188 grams of agar, 262 grams of inactive yeast, 930 grams of maize flour, 120 grams of soy flour, 1400 milliliters of molasses, 140 milliliters of Tegosept solution, 50 milliliters of propionic acid, and 10 milliliters of phosphoric acid. Flies were kept in 12 h light: 12 h dark cycles (LD) at 25°C (ZT 0 is the beginning of the light phase at 8:00 AM, ZT 12 is the beginning of the dark phase at 8:00 PM) except for some experimental manipulation. For temperature-controlled experiments, including those utilizing the temperature-sensitive tub-GAL80^ts^ driver, the flies were initially crossed and maintained at a constant temperature of 22°C within an incubator. The temperature shift was initiated post-eclosion. Once the flies had emerged, they were transferred to an incubator set at an elevated temperature of 29°C for a defined period, after which the experimental protocols were carried out. Wild-type flies were *Canton-S* (*CS*). The genotypes of the flies used in each figure were listed in Table 1.

**Table 1:** Genotypes of flies used for experiments in this study

Figure panel	Genotype
Figure 1A	*GAL4*^*SIFa. PT*^*/UAS-GFP. nls* *GAL4*^*SIFa. PT*^*/UAS-TNT* *GAL4*^*SIFa. PT*^*/UAS-KCNJ2* *GAL4*^*SIFa. PT*^*/UAS-OrkΔC* *GAL4*^*SIFa. PT*^*/UAS-NaChBac*
Figure 1B	*GAL4*^*empty*^*/SIFa-RNAi* *GAL4*^*SIFa. PT*^*/empty-RNAi* *GAL4*^*SIFa. PT*^*/SIFa-RNAi*
Figure 1C	Same as Figure 1A
Figure 1D	Same as Figure 1B
Figure 1E	*GAL4*^*SIFa. PT*^*/empty-RNAi* *GAL4*^*SIFa. PT*^*/Chat-RNAi* *GAL4*^*SIFa. PT*^*/Gad1-RNAi* *GAL4*^*SIFa. PT*^*/VGlut-RNAi* *GAL4*^*SIFa. PT*^*/ple-RNAi* *GAL4*^*SIFa. PT*^*/Tdc2-RNAi* *GAL4*^*SIFa. PT*^*/Trhn-RNAi*
Figure 1F	Same as Figure 1E
Figure 1G	*GAL4*^*SIFa. PT*^*/empty-RNAi* *GAL4*^*SIFa. PT*^*/CrzR-RNAi* *GAL4*^*SIFa. PT*^*/CCHa1-R-RNAi* *GAL4*^*SIFa. PT*^*/InR-RNAi* *GAL4*^*SIFa. PT*^*/Lgr3-RNAi* *GAL4*^*SIFa. PT*^*/Lgr4-RNAi* *GAL4*^*SIFa. PT*^*/PK2-R1-RNAi* *GAL4*^*SIFa. PT*^*/PK2-R2-RNAi* *GAL4*^*SIFa. PT*^*/CCKLR17D1-RNAi* *GAL4*^*SIFa. PT*^*/CCKLR17D3-RNAi* *GAL4*^*SIFa. PT*^*/sNPR-R-RNAi*
Figure 1H	Same as Figure 1G
Figure 2A	*GAL4*^*empty*^*/SIFaR-RNAi repo-GAL4/SIFaR-RNAi* *nSyb-GAL4/SIFaR-RNAi*
Figure 2B	Same as Figure 2A
Figure 2C	*fru-GAL4/empty-RNAi* *fru-GAL4/SIFaR-RNAi* *dsx-GAL4/empty-RNAi* *dsx-GAL4/SIFaR-RNAi*
Figure 2D	Same as Figure 2C
Figure 2E	*GAL4*^*empty*^*/UAS-TNT, tub-GAL80*^*ts*^ *SIFaR*^*24F06*^*/UAS-TNT, tub-GAL80*^*ts*^ *SIFaR*^*2A*^*/UAS-GFP* *SIFaR*^*2A*^*/UAS-TNT, tub-GAL80*^*ts*^ *GAL4*^*empty*^*/UAS-stop-TNT, fru*^*FLP*^ *SIFaR*^*2A*^*/UAS-stop-TNT, fru*^*FLP*^
Figure 2F	Same as Figure 2E
Figure 3A	*fru-GAL4/SIFaR-RNAi*
Figure 3B	*fru-GAL4/empty-RNAi*
Figure 3C	*fru-GAL4/AstC-R1-RNAi*
Figure 3C	*fru-GAL4/AstC-R2-RNAi*
Figure 3E	*elav*^*c155*^*/SIFaR-RNAi* *elav*^*c155*^*/AstC-RNAi* *elav*^*c155*^*/SIFa-RNAi*
Figure 3F	*elav*^*c155*^*/empty-RNAi* *elav*^*c155*^*/AstC-R1-RNAi* *elav*^*c155*^*/AstC-R2-RNAi* *elav*^*c155*^*/SIFaR-RNAi*
Figure 3G	*SIFaR*^*2A*^*-GAL4, fru-lexA/UAS-Stinger*, *lexAop-tdTomato.nls*
Figure 4B	*SIFaR*^*R23G06*^*/UAS-stop-TNT, fru*^*FLP*^
Figure 4C	*SIFaR*^*R24A12*^*/UAS-stop-TNT, fru*^*FLP*^
Figure 4D	*SIFaR*^*R24F06*^*/UAS-stop-TNT, fru*^*FLP*^
Figure 4E	*SIFaR*^*R57F10*^*/UAS-stop-TNT, fru*^*FLP*^
Figure 4F	*GAL4*^*empty*^*/UAS-stop-TNT, fru*^*FLP*^ *SIFaR*^*R23G06*^*/UAS-stop-TNT, fru*^*FLP*^ *SIFaR*^*R24A12*^*/UAS-stop-TNT, fru*^*FLP*^ *SIFaR*^*R24F06*^*/UAS-stop-TNT, fru*^*FLP*^ *SIFaR*^*R57F10*^*/UAS-stop-TNT, fru*^*FLP*^
Figures 4G–4L	*SIFaR*^*R57F10*^*, fru*^*FLP*^*/UAS-stop-mCD8GFP, UAS-RedStinger*
Figure 5A	*SIFaR*^*2A*^*, fru*^*FLP*^*/UAS-stop-mCD8GFP*
Figure 5B	*SIFaR*^*2A*^*, dsx*^*FLP*^*/UAS-stop-mCD8GFP*
Figure 5C	*fruP1-GAL4/SIFaR-RNAi*
Figure 5D	*GAL4*^*R43D01*^*/SIFaR-RNAi*
Figure 5E	*fruP1-GAL4/empty-RNAi*
Figure 5F	*GAL4*^*R43D01*^*/empty-RNAi*
Figures 5G and 5H	*GAL4*^*fruP1*^*/lexAop-CD2-GFP, UAS-mLexA-VP16-NFAT, lexAop-CD8-GFP-A2-CD8-GFP*
Figures 5J and 5K	*fruP1-GAL4/empty-RNAi* *fruP1-GAL4/SIFaR-RNAi* *GAL4*^*R43D01*^*/empty-RNAi* *GAL4*^*R43D01*^*/SIFaR-RNAi*
Figures 6A and 6B	*SIFaR*^*2A*^*, fru*^*FLP*^*/UAS-stop-Dscam-GFP*
Figures 6C and 6D	*SIFaR*^*2A*^*, fru*^*FLP*^*/UAS-stop-nSyb-GFP*
Figures 6E and 6F	*SIFa*^*lexA.PT*^*, GAL4*^*R43D01*^*/UAS-post-t-GRASP, lexAop2-pre-t-GRASP*
Figures 6G–6I	*SIFa*^*lexA.PT*^*, GAL4*^*R43D01*^*/lexAop-CsChrimson, UAS-GCaMP7s*
Figures S2A–S2C	*GAL4*^*empty*^*/UAS-stop-TNT, fru*^*FLP*^ *SIFaR*^*2A*^*/UAS-stop-TNT, fru*^*FLP*^
Figures S3A–S3D	*GAL4*^*empty*^*/AstC-R1-RNAi* *GAL4*^*empty*^*/AstC-R2-RNAi dsx-GAL4/empty-RNAi* *GAL4*^*empty*^*/SIFaR-RNAi*
Figures S4A–S4F	*GAL4*^*empty*^*/UAS-stop-TNT, fru*^*FLP*^ *SIFaR*^*R23G06*^*/UAS-stop-TNT, fru*^*FLP*^ *SIFaR*^*R24A12*^*/UAS-stop-TNT, fru*^*FLP*^ *SIFaR*^*R24F06*^*/UAS-stop-TNT, fru*^*FLP*^ *SIFaR*^*R57F10*^*/UAS-stop-TNT, fru*^*FLP*^
Figure S4G	*SIFaR*^*R57F10*^*, fru*^*FLP*^*/UAS-stop-mCD8GFP, UAS-RedStinger*
Figure S5A	*fruP1-GAL4, SIFaR*^*R24F06*^*-lexA/UAS-mCD8RFP, lexAop-mCD8GFP*
Figure S5B	*fruP1-GAL4, SIFaR*^*T2A*^*-lexA/UAS-mCD8RFP, lexAop-mCD8GFP*
Figure S5C	*fruP1-GAL4, Tsh-GAL80/SIFaR-RNAi*
Figures S5D and S5E	*fruP1-GAL4, Tsh-GAL80/empty-RNAi* *fruP1-GAL4, Tsh-GAL80/SIFaR-RNAi*
Figure S5F	*GAL4*^*R43D01*^*, SIFaR*^*R24F06*^*-lexA/UAS-mCD8RFP, lexAop-mCD8GFP*
Figure S5G	*GAL4*^*R43D01*^*/lexAop-CD2-GFP, UAS-mLexA-VP16-NFAT, lexAop-CD8-GFP-A2-CD8-GFP*
Figures S5H and S5I	*Tk-GAL4/SIFaR-RNAi*
Figures S5J and S5K	*SIFaR-GAL4/Tk-RNAi*
Figure S6A	*SIFaR*^*2A*^*-lexA, GAL4*^*R43D01*^*/UAS-Stinger*, *lexAop-tdTomato.nls*
Figure S6B	*GAL4*^*SIFa.PT*^*, lexA*^*SIFa.PT*^*/UAS-mCD8RFP, lexAop-mCD8GFP*

#### Validation of RNAi lines

All RNAi lines used in this study were selected based on prior validation of their efficacy and specificity in published work. This includes all lines targeting *SIFa-RNAi*,^12^ *SIFaR-RNAi*,^89^ *Chat-RNAi*,^26^ *VGlut-RNAi*,^26^ *CrzR-RNAi*,^90^ *ple-RNAi*,^27^ *Gad1-RNAi*,^28^ *Tdc2-RNAi*,^29^ *Trhn-RNAi*,^26^ *Lgr4-RNAi*,^91^ *Lgr3-RNAi*,^91^ *CCKLR-17D3-RNAi*,^92^ *CCKLR-17D1-RNAi*,^92^ *sNPFR-RNAi*,^92^ *Tk-RNAi*,^90^ *AstC-R2-RNAi*,^53^ *CCHa1-R-RNAi*,^93^ *InR-RNAi*,^94^ *PK2-R1-RNAi*,^95^ *PK2-R2-RNAi*,^96^ and *AstC-R1-RNAi*.^51^ These independent studies confirmed target gene knockdown using methods including qRT-PCR, Western blotting, and/or phenotypic rescue with multiple RNAi strains. The control genotype (*empty-RNAi*^97^) was similarly validated to lack non-specific effects.

#### Courtship assays for copulation latency (CL) and courtship index (CI)

Courtship assay was performed as previously described^103^ under normal light conditions in circular courtship arenas 11 mm in diameter, from noon to 4 pm. We utilized the time at which copulation (mating) was initiated to quantify copulation latency (CL). The statistical methodology applied for the analysis of CL is identical to that employed for the MD assay. Upon the onset of courtship behavior, the courtship index (CI) was computed as the proportion of time a male dedicated to courtship-related behaviors within a 10-minute observation window or until the initiation of mating. The initiation of mating is defined as the moment at which male flies successfully achieve mounting on females. All copulation latency (CL) and mating behavior assays were performed using *SPR*^*-/-*^ null mutant females, *Df(1)*^*exel6234*^ to standardize female receptivity.^24^ This approach eliminates variation from female rejection behaviors, constitutively elevates baseline receptivity (>95% acceptance rate, and enables isolation of male-specific contributions to CL.

#### Mating duration assays

The mating duration assay in this study has been reported.^38^^,^^39^^,^^48^ To enhance the efficiency of the mating duration assay, we utilized the *Df(1)*^*Exel6234*^ (DF here after) genetic modified fly line in this study, which harbors a deletion of a specific genomic region that includes the sex peptide receptor (SPR).^24^^,^^104^ Previous studies have demonstrated that virgin females of this line exhibit increased receptivity to males.^24^ We conducted a comparative analysis between the virgin females of this line and the CS virgin females and found that both groups induced SMD. Consequently, we have elected to employ virgin females from this modified line in all subsequent studies. For group reared (naïve) males, 40 males from the same strain were placed into a vial with food for 5 days. For single reared males, males of the same strain were collected individually and placed into vials with food for 5 days. For experienced males, 40 males from the same strain were placed into a vial with food for 4 days then 80 DF virgin females were introduced into vials for last 1 day before assay. 40 DF virgin females were collected from bottles and placed into a vial for 5 days. These females provide both sexually experienced partners and mating partners for mating duration assays. At the fifth day after eclosion, males of the appropriate strain and DF virgin females were mildly anaesthetized by CO_2_. After placing a single female into the mating chamber, we inserted a transparent film then placed a single male to the other side of the film in each chamber. After allowing for 1 h of recovery in the mating chamber in 25°C incubators, we removed the transparent film and recorded the mating activities. Only those males that succeeded to mate within 1 h were included for analyses. Initiation and completion of copulation were recorded with an accuracy of 10 sec, and total mating duration was calculated for each couple.^12^^,^^39^^,^^89^^,^^91^^,^^92^ Genetic controls for LMD and SMD behaviors were incorporated exclusively when assessing novel fly strains that had not previously been examined. In essence, internal controls were predominantly employed in the experiments, as LMD and SMD behaviors exhibit enhanced statistical significance when internally controlled. Within the LMD assay, both group and single conditions function reciprocally as internal controls. A significant distinction between the naïve and single conditions implies that the experimental manipulation does not affect LMD. Conversely, the lack of a significant discrepancy suggests that the manipulation does influence LMD. In the context of SMD experiments, the naïve condition (equivalent to the group condition in the LMD assay) and sexually experienced males act as mutual internal controls for one another. A statistically significant divergence between naïve and experienced males indicates that the experimental procedure does not alter SMD. Conversely, the absence of a statistically significant difference suggests that the manipulation does impact SMD. Hence, we incorporated supplementary genetic control experiments solely if they deemed indispensable for testing. All assays were performed from noon to 4 PM. We conducted blinded studies for every test.

#### Quantification and interpretation of mating duration behaviors

Long Mating Duration (LMD): Males exposed to rivals exhibit significantly prolonged MD compared to socially naive control males (no rival exposure). Intact LMD is defined as a statistically significant increase (p < 0.05) in MD in rival-exposed males versus controls.^38^^,^^39^^,^^43^^,^^44^^,^^46^

Short Mating Duration (SMD): Sexually experienced males exhibit significantly reduced MD compared to sexually naive control males. Intact SMD is defined as a statistically significant decrease (p < 0.05) in MD in experienced males versus controls.^47^^,^^48^^,^^105^^,^^106^^,^^107^

Intact Behavior Criteria: LMD or SMD is considered *intact* when rival-exposed males show a statistically significant increase (p < 0.05) in MD versus controls (LMD), *or* experienced males show a statistically significant decrease (p < 0.05) in MD versus controls (SMD).

Abolished Behavior Criteria: LMD or SMD is considered *abolished* when experimental manipulations eliminate the adaptive duration difference, resulting in no statistically significant difference (p ≥ 0.05) in MD between rival-exposed males versus controls (LMD), *or* experienced males versus controls (SMD).

Note: “Abolished” indicates loss of context-dependent mating duration modulation; mating initiation and completion persist in all groups.

#### Generation of transgenic flies

To generate the *SIFa*^*PT*^*-lexA* driver, the putative promotor sequence of the gene was amplified by PCR using wild-type genomic DNA as a template with the following primers GCCAATTGGCTGAATCTCCTGACCCTCA and GCAGATCTCTTGCAGTTTTCGGTGAGC as mentioned before.^15^ The amplified DNA fragment (1482 base pairs located immediately upstream of the *SIFa* coding sequence) was inserted into the E2 Enhancer-lexA vector. This vector, supplied by Qidong Fungene Biotechnology Co., Ltd. (http://www.fungene.tech/), is a derivative of the pBPLexA::p65Uw vector (available at https://www.addgene.org/26231). The insertion was achieved by digesting the fragment and the vector with EcoRI and XbaI restriction enzymes to create compatible sticky ends. The genetic construct was inserted into the *attp2* site on chromosome III to generate transgenic flies using established techniques, a service conducted by Qidong Fungene Biotechnology Co., Ltd.

#### Immunostaining

The dissection and immunostaining protocols for the experiments are described elsewhere.^38^ After 5 days of eclosion, the *Drosophila* brain was taken from adult flies and fixed in 4% formaldehyde at room temperature for 30 minutes. The sample was washed three times (5 minutes each) in 1% PBT and then blocked in 5% normal goat serum for 30 minutes. The sample next be incubated overnight at 4°C with primary antibodies in 1% PBT, followed by the addition of fluorophore-conjugated secondary antibodies for one hour at room temperature. The brain was mounted on plates with an antifade mounting solution (Solarbio) for imaging purposes.

Confocal imaging was conducted utilizing a ZEISS LSM880 microscope. GFP detection was accomplished through immunostaining with chicken anti-GFP primary antibody (1:500; A10262, Invitrogen) and Alexa Fluor 488-conjugated donkey anti-chicken IgG secondary antibody (1:200, Jackson ImmunoResearch), facilitating the visualization of: (i) native GFP expression, (ii) CalexA GFP signals, and (iii) t-GRASP reconstitution GFP signals based on previous studies.^108^^,^^109^^,^^110^ For anatomical reference and supplementary labeling, the following antibodies were used: (i) mouse anti-Bruchpilot (nc82; 1:50, DSHB) detected by Alexa Fluor 647-conjugated goat anti-mouse IgG (1:200, Jackson ImmunoResearch) to accentuate neuropil architecture, and (ii) rabbit anti-RFP (1:500, Rockland Immunochemicals) with Alexa Fluor 555-conjugated goat anti-rabbit IgG (1:200, Invitrogen) for RFP detection.

#### Quantitative analysis of fluorescence intensity

To ascertain calcium levels and synaptic intensity from microscopic images, we dissected and imaged five-day-old flies of various social conditions and genotypes under uniform conditions. For group reared (naïve) flies, the flies were reared in group condition and dissect right after 5 days of rearing without any further action. For single reared flies, the flies were reared in single condition and dissect at the same time as group reared flies right after 5 days of rearing without any further action. For sexual experienced flies, the flies were reared in group condition after 4 days of rearing and will be given virgins to give them sexual experience for one day, those flies will also be dissected at the same time as group and single reared flies after one day. The GFP signal in the brains and VNCs was amplified through immunostaining with chicken anti-GFP primary antibody. Image analysis was conducted using ImageJ software. For CaLexA signal quantification, we adhered to protocols detailed by Kayser et al.,^111^ which involve measuring the ROI's GFP-labeled area by summing pixel values across the image stack. This method assumes that changes in the GFP-labeled area are indicative of alterations in the CaLexA signal, reflecting synaptic activity. ROI intensities were background-corrected by measuring and subtracting the fluorescent intensity from a non-specific adjacent area, as per Kayser et al.^111^ For the analysis of GRASP or tGRASP signals, a sub-stack encompassing all synaptic puncta was thresholded by a genotype-blinded investigator to achieve the optimal signal-to-noise ratio. The fluorescence area or ROI for each region was quantified using ImageJ, employing a similar approach to that used for CaLexA quantification.^112^

#### Colocalization analysis

Before the colocalization analysis, an investigator, blinded to the fly's genotype, thresholded the sum of all pixel intensities within a sub-stack to optimize the signal-to-noise ratio, following established methods.^112^ To perform colocalization analysis of multi-color fluorescence microscopy images in this study, we employed ImageJ software.^113^ In brief, we merged image channels to form a composite with accurate color representation and applied a threshold to isolate yellow pixels, signifying colocalization. The measured “area” values represented the colocalization zones between fluorophores. To determine the colocalization percentage relative to the total area of interest (e.g., GFP), we adjusted thresholds to capture the full fluorophore areas and remeasured to obtain total areas. The quantification of the overlap was performed using confocal images with projection by standard deviation function provided by ImageJ to ensure precise measurements and avoid pixel saturation artifacts. The colocalization efficiency was calculated by dividing the colocalized area by the total fluorophore area. All samples were imaged uniformly.

#### Particle analysis

Before the particle analysis, an investigator, blinded to the fly's genotype, thresholded the sum of all pixel intensities within a sub-stack to optimize the signal-to-noise ratio, following established methods.^112^ To quantitatively measure particle intensity of cell number and synaptic puncta in microscopic images, we applied ImageJ software. Initially, the image is converted to grayscale to reduce complexity and enhance contrast. Subsequently, thresholding techniques are employed to binarize the image, distinguishing particles from the background. This binarization can be achieved through automated thresholding algorithms or manual adjustment to optimize the segmentation. The results of these measurements are then available for review by conducting "Analyze Particles" function of ImageJ. All specimens were imaged under identical conditions.

#### Climbing assay

For climbing assay, we modified the conventional RING assay.^114^^,^^115^ In brief, 40-50 20-day-aged flies were placed in an empty vial and were tapped to the bottom of the tube. After tapping of flies, we recorded 10 seconds of video clip. This experiment was done five times with 5-minute intervals. With recorded video files, we captured the position of flies 10 seconds after tapping the vial. This captured image file was then loaded in ImageJ to perform particle analysis. For quantifying the location of flies inside a vial, we used the “analyze particles” function of ImageJ.^116^ The position of pixels was normalized by height of vial then only the particles above the midline (4 cm) of vial were counted.

#### Feeding of retinal

All trans-retinal powder (Sigma) was dissolved in EtOH as a 100 mM stock solution. 200 μl of this stock solution was diluted in 50 mL of melted normal food to prepare 400 μM of all trans-retinal (ATR) food. For CsChrimson experiments, male flies of proper genotype were selected and separated into two groups (with and without ATR) after 5 days of eclosion for at least 3 days prior to any optogenetic experiments.

#### Two-photon calcium imaging

To measure the neuron activity in AG region, the flies were carefully sedated with ice and then placed in an inverted posture on a plastic plate coated with UV glue, which securely held flies in the middle. Whole fly was exposed to AHL for red light activation. The plate was filled with *Drosophila* Adult Hemolymph-Like Saline (AHLS) buffer [108 mM NaCl, 5 mM KCl, 4 mM NaHCO_3_, 1 mM NaH_2_PO_4_, 15 mM ribose, 5 mM Hepes (pH 7.5), 300 mosM, CaCl_2_ (2 mM), and MgCl_2_ (8.2 mM)] to ensure the flies’ neurons remained in an active state throughout the experiment.^26^^,^^117^ Subsequently, the flies were dissected to specifically expose the VNC for detailed examination. Calcium imaging was performed using Zeiss LSM880 microscope with a 20x water immersion objective. Images were acquired at 2 frames per second at a resolution of 512 × 512 pixels. GCaMP7 slow (GCaMP7s) signals were recorded with an 880 nm laser and optogenetic stimulation was achieved with a 590-595 nm 20 W red light pulses at 50-60 Hz (0.16 mW/mm^2^), LED stimulation last 2 s. ROIs were manually selected from the cell body in AG area with ImageJ.

*ΔF*/*F*_*0*_ = (*F*_*t*_ − *F*_*0*_)/*F*_*0*_ × 100% and *Peak ΔF*/*F*_*0*_ = (*Peak F*_*t*_–*F*_*0*_)/*F*_*0*_ × 100% were used to determine the fluorescence change, where Ft is the fluorescence at time point n and *F*_*0*_ is the fluorescence from the average intensity of 10 frames fluorescence before optogenetic stimulation. To quantify neuronal activity in the brain region, the brains of male flies with the appropriate genotype were dissected and secured in a plastic dish. All subsequent steps were conducted as previously described.^26^^,^^117^

#### scRNA-seq data analysis

We utilized the single-cell RNA-seq data from https://flycellatlas.org,^118^ and the UMIs (Unique Molecular Identifiers) data are generated from the loom file of 10x VSN All (Stringent), which comprises a total of 506,660 cells. The data were subsequently analyzed by Seurat (v4.3.0).^50^ And we used the 'NormalizeData' function to automatically normalize the data. The Dot plot is based on the function 'Dotplot', the cells were first divided according to the 'annotation' which refers to the cell type, then the table of dot plot of one gene was calculated by the function 'Dotplot', and finally, the organization category was placed on the x-axis.

#### Statistical tests

Statistical analysis of mating duration assay was described previously.^38^^,^^39^^,^^48^ More than 50 males (naïve, experienced and single) were used for mating duration assay. Our experience suggests that the relative mating duration differences between naïve and experienced condition and singly reared are always consistent; however, both absolute values and the magnitude of the difference in each strain can vary. So, we always include internal controls for each treatment as suggested by previous studies.^119^ Therefore, statistical comparisons were made between groups that were naïvely reared, sexually experienced and singly reared within each experiment. As mating duration of males showed normal distribution (Kolmogorov-Smirnov tests, p > 0.05), we used two-sided Student’s *t*-tests. As mating duration of males showed normal distribution (Kolmogorov-Smirnov tests, p > 0.05), we used two-sided Student’s t tests. The mean ± standard error (s.e.m) (*∗∗∗∗ = p < 0.0001, ∗∗∗ = p < 0.001, ∗∗ = p < 0.01, ∗ = p < 0.05*). All analysis was done in GraphPad (Prism). Individual tests and significance are detailed in figure legends.

Besides traditional *t*-test for statistical analysis, we added estimation statistics for all MD assays and two group comparing graphs. In short, ‘estimation statistics’ is a simple framework that—while avoiding the pitfalls of significance testing—uses familiar statistical concepts: means, mean differences, and error bars. More importantly, it focuses on the effect size of one’s experiment/intervention, as opposed to significance testing.^120^ In comparison to typical NHST plots, estimation graphics have the following five significant advantages such as (1) avoid false dichotomy, (2) display all observed values (3) visualize estimate precision (4) show mean difference distribution. And most importantly (5) by focusing attention on an effect size, the difference diagram encourages quantitative reasoning about the system under study.^121^ Thus, we conducted a reanalysis of all our two group data sets using both standard *t* tests and estimate statistics. In 2019, the Society for Neuroscience journal eNeuro instituted a policy recommending the use of estimation graphics as the preferred method for data presentation.^122^

For comparisons involving three or more experimental groups (e.g., CI and CL measurements), we conducted one-way analysis of variance (ANOVA). When ANOVA indicated significant main effects (p < 0.05), we performed Tukey's honestly significant difference (HSD) post hoc tests to control for multiple comparisons while maintaining the family-wise error rate at α = 0.05. The effect size for the overall ANOVA model was quantified using the coefficient of determination (R^2^), representing the proportion of variance in the dependent variable explained by group differences. We interpreted R^2^ values using Cohen's conventional thresholds: 0.01 ≤ R^2^ < 0.09 (small effect), 0.09 ≤ R^2^ < 0.25 (medium effect), and R^2^ ≥ 0.25 (large effect).

### Quantification and statistical analysis

All statistical analyses were performed using GraphPad Prism 10 unless otherwise indicated. The statistical details for each experiment, including the test used, sample size (n), definition of n, and measures of central tendency and variability, are provided in the figure legends. In general, n represents the number of animals (flies) tested for behavioral assays (mating duration assays and courtship assays) and the number of cells (neurons) analyzed for neuronal morphology quantification. Data are expressed as mean ± SEM unless otherwise stated. DBM (Difference Between Means) plots show the mean difference with 95% confidence intervals estimated by bootstrap resampling. For pairwise comparisons, two-tailed Student’s t-tests were used; for multiple group comparisons, one-way ANOVA followed by Tukey’s post hoc test was applied. All tests assumed normal distribution of the data (Kolmogorov–Smirnov test). Exact statistical test used in each assay are indicated in each figure legend.
