# Supplementary material for: SIFamide-dependent synaptic plasticity in male-specific GABAergic neurons underlies experience-modulated mating behaviors
Source: iScience. 2025 Sep 5;28(10):113516. doi: 10.1016/j.isci.2025.113516 (PMC12483593; doi:10.1016/j.isci.2025.113516)
Supplement: Document S1. Figures S1–S6 [file mmc1.pdf]

iScience, Volume *28*

Supplemental information

SIFamide-dependent synaptic plasticity in male-specific GABAergic neurons underlies experience-modulated mating behaviors

Tianmu Zhang (张天目), Hongyu Miao (苗鸿钰), Yutong Song (宋雨桐), Zekun Wu (吴泽坤), and Woo Jae Kim (金佑宰김우재)

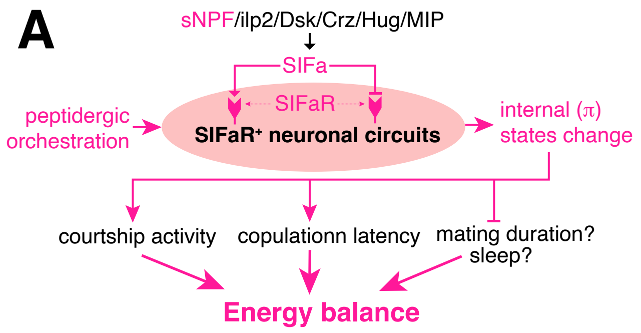

SIFa-Fru, Fig. S1

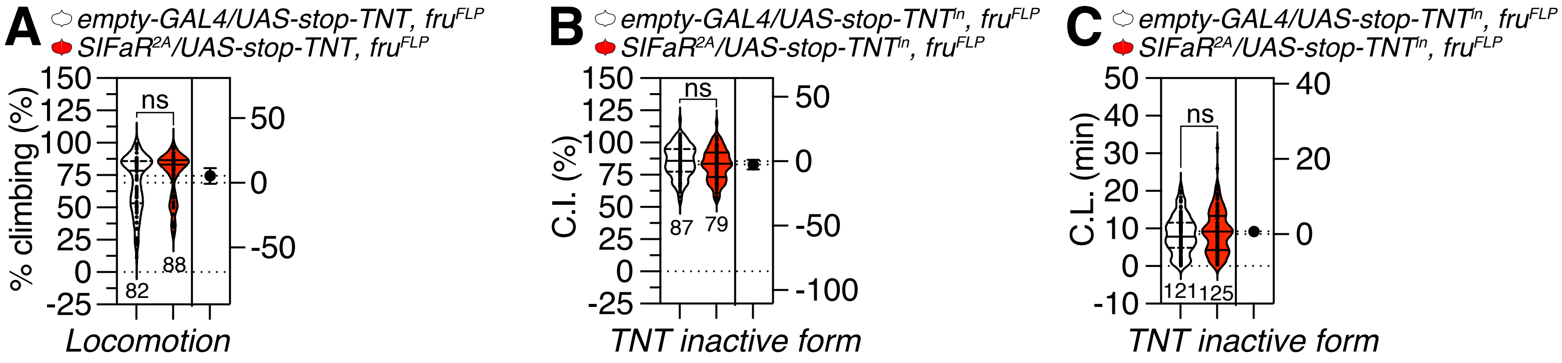

SIFa-Fru, Fig. S2

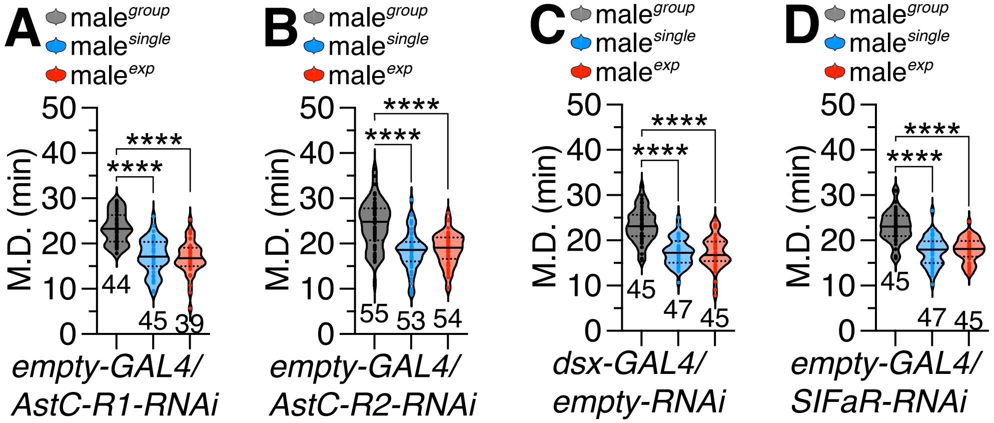

SIFa-Fru, Fig.S3

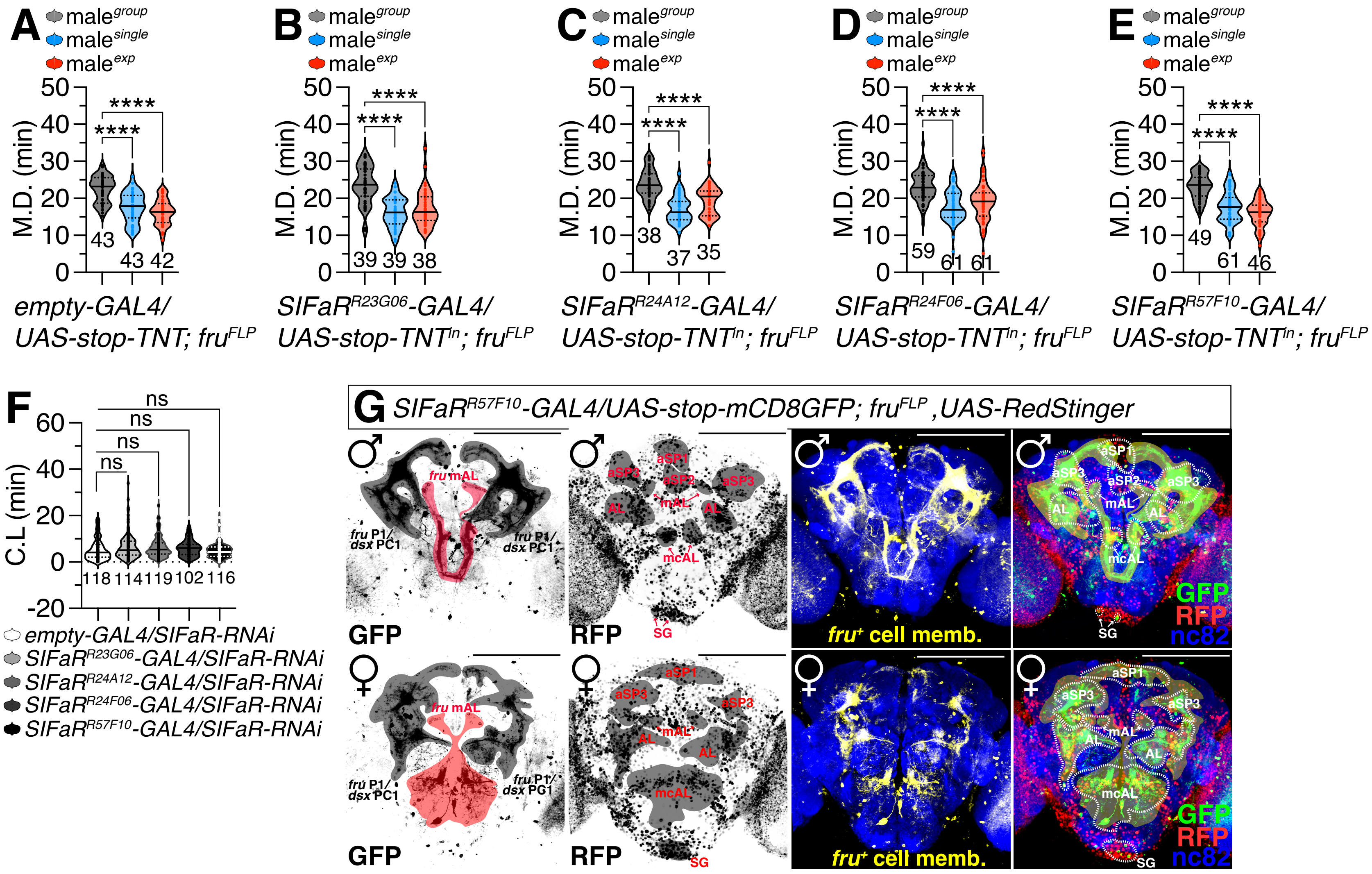

SIFa-Fru, Fig. S4

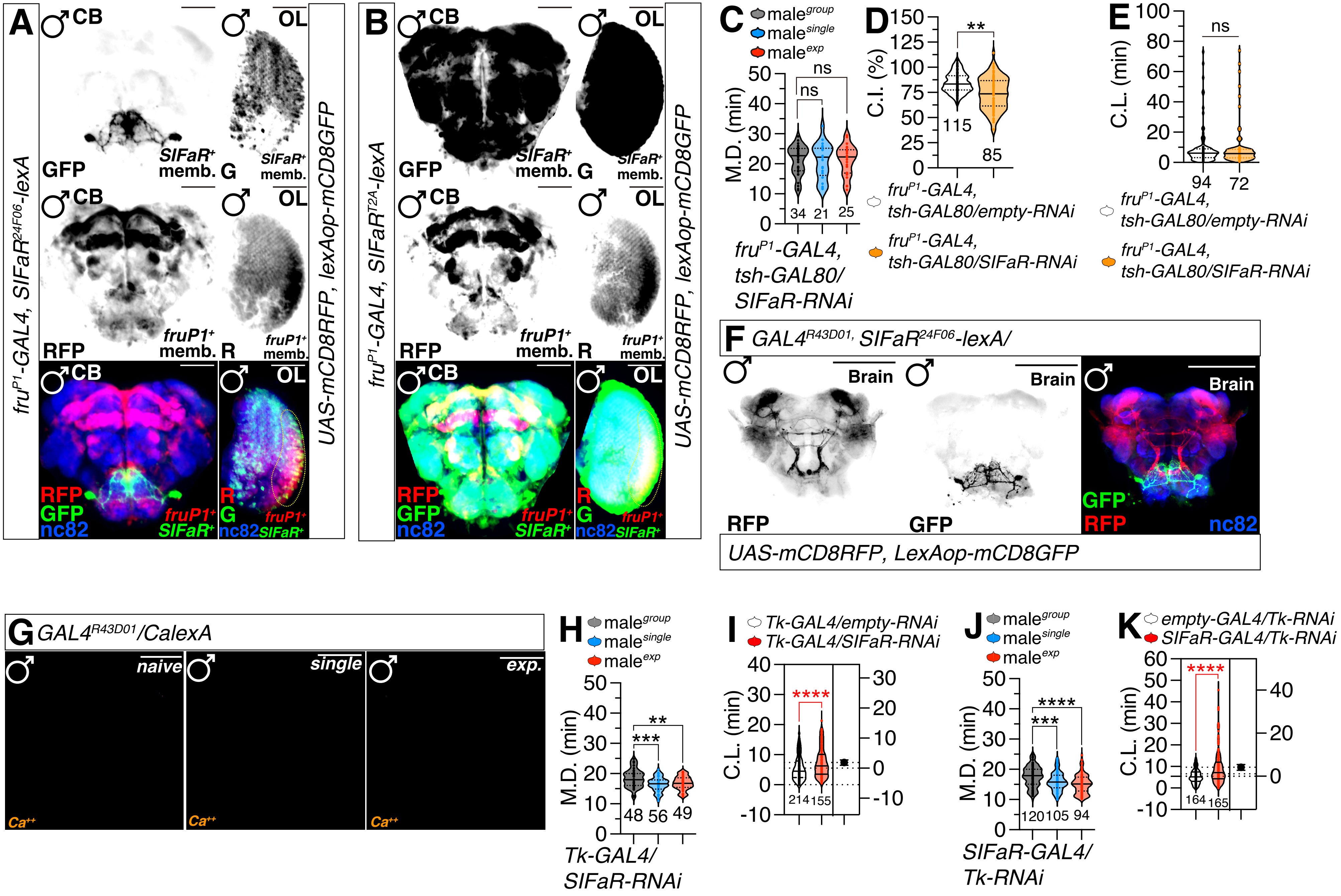

SIFa-Fru, Fig.S5

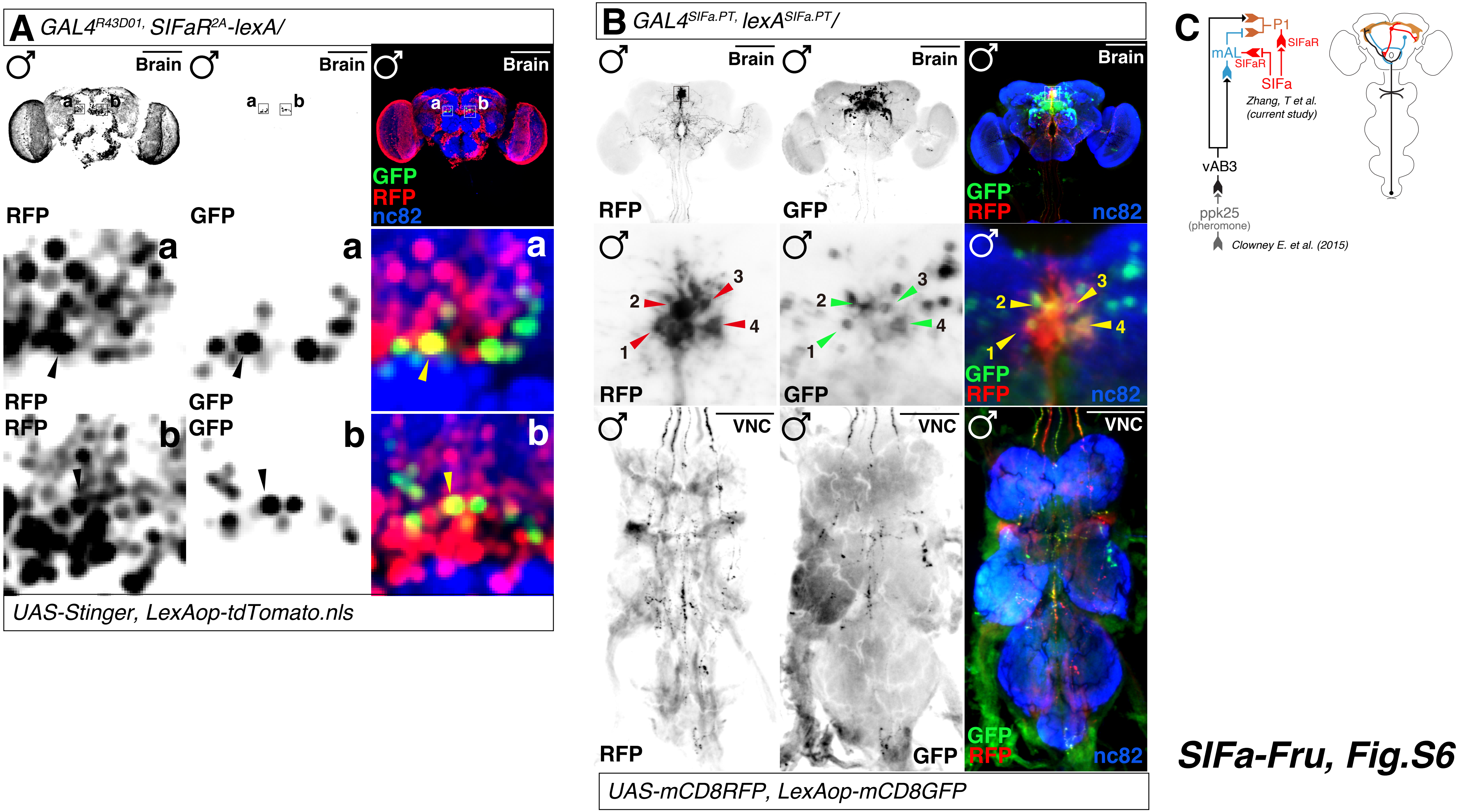

SIFa-Fru, Fig.S6

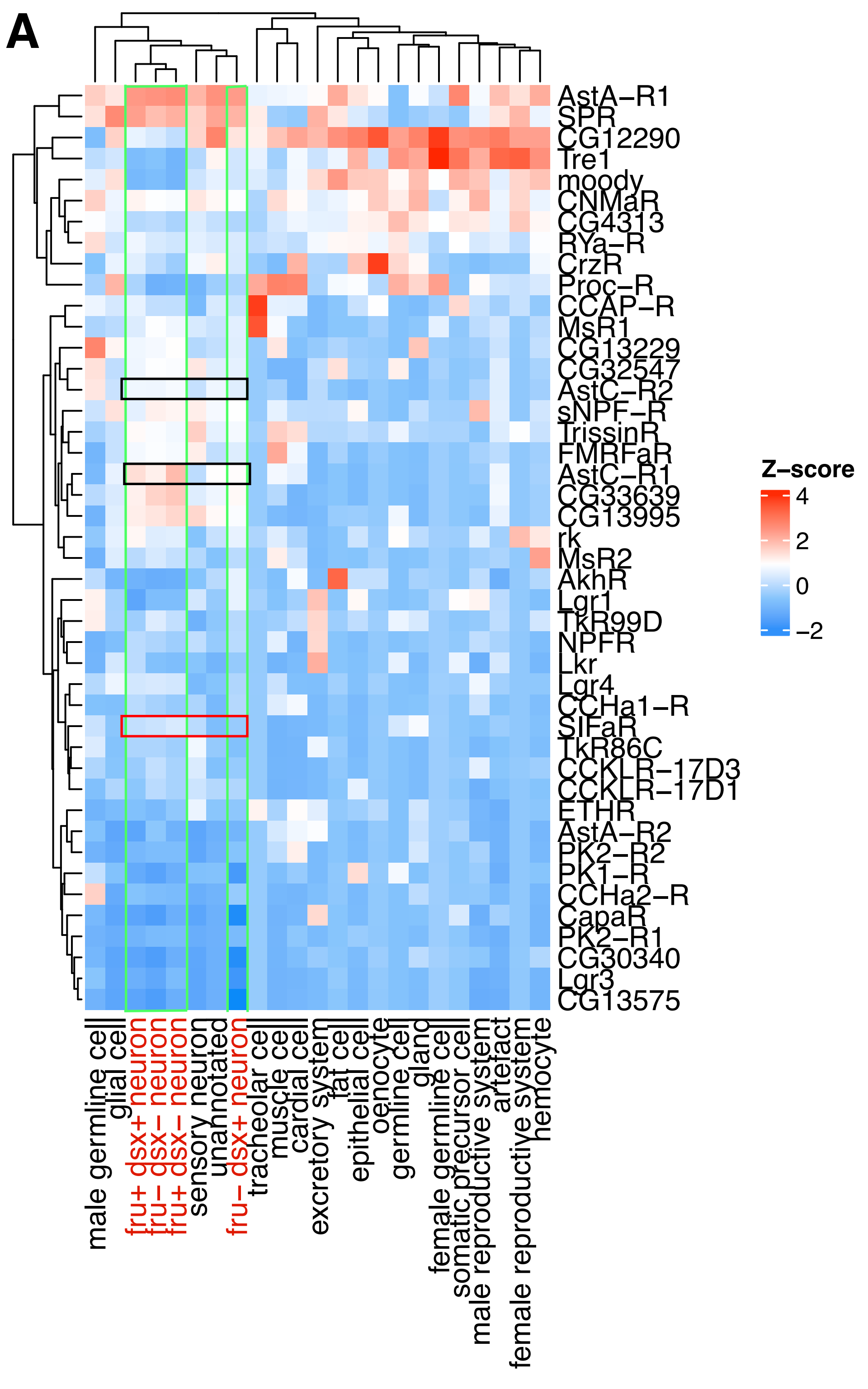
A
AstA−R1
SPR
CG12290
Tre1
moody
CNMaR
CG4313
RYa−R
CrzR
Proc−R
CCAP−R
MsR1
CG13229
CG32547
AstC−R2
sNPF−R
TrissinR
FMRFaR
AstC−R1
CG33639
CG13995
rk
MsR2
AkhR
Lgr1
TkR99D
NPFR
Lkr
Lgr4
CCHa1−R
SIFaR
TkR86C
CCKLR−17D3
CCKLR−17D1
ETHR
AstA−R2
PK2−R2
PK1−R
CCHa2−R
CapaR
PK2−R1
CG30340
Lgr3
CG13575
Z−score
4
2
0
−2
male germline cell
glial cell
fru+ dsx+ neuron
fru− dsx− neuron
fru+ dsx− neuron
sensory neuron
unannotated
fru− dsx+ neuron
tracheolar cell
muscle cell
cardial cell
excretory system
fat cell
epithelial cell
oenocyte
germline cell
gland
female germline cell
somatic precursor cell
male reproductive system
artefact
female reproductive system
hemocyte

SUPPLEMENTAL FIGURE TITLES AND LEGENDS

Fig. S1 SIFaR-expressing neurons integrate diverse neuropeptide inputs and regulate sexual behaviors.

(A) Diagram of functional connectivity between SIFa and SIFaR neurons

Fig. S2 SIFaR-positive neurons are crucial for male sexual behavior and are not involved in locomotion.

(A) Climbing assay of males expressing *empty-GAL4* and $SIFaR^{2A}$ with *UAS-stop-TNT,* fru^{FLP} (two-tailed unpaired *t*-test). In all plots and statistical tests. Data are presented as mean ± s.e.m. ns = not significant ($p>0.05$), *$*p<0.05$, $**p<0.01$, $***p< 0.001$, $****p< 0.0001$*. Sample sizes (n) are indicated in the figure panels. Data are represented as mean +/- SEM.

(B-C) CI and CL of group-housed males expressing *empty-GAL4* and $SIFaR^{2A}$ together with $UAS\text{-}stop\text{-}TNT^{ts}; fru^{FLP}$ (two-tailed unpaired *t*-test). In all plots and statistical tests. Data are presented as mean ± s.e.m. ns = not significant ($p>0.05$), $*p<0.05$, $**p<0.01$, $***p< 0.001$, $****p< 0.0001$. Sample sizes (n) are indicated in the figure panels. Data are represented as mean +/- SEM.

Fig. S3 SIFaR and AstC signaling in fru-positive neurons regulate mating duration.

(A) MD assays of flies expressing the *empty-GAL4* driver together with *AstC-R1-RNAi*. Statistical significance determined by one-way ANOVA followed by Tukey’s multiple comparisons test. $p < 0.0001$ (one-way ANOVA, $F = 44.09$, $R^2 = 0.41$; Tukey's post

hoc). Sample sizes (n) are indicated in the figure panels. Data are represented as mean +/- SEM.

(B) MD assays of flies expressing the *empty-GAL4* driver together with *AstC-R2-RNAi*. Statistical significance determined by one-way ANOVA followed by Tukey’s multiple comparisons test. $p < 0.0001$ (one-way ANOVA, $F = 29.31$, $R^2 = 0.27$; Tukey's post hoc). Sample sizes (n) are indicated in the figure panels. Data are represented as mean +/- SEM.

(C) MD assays of flies expressing the *dsx-GAL4* driver together with *empty-RNAi*. Statistical significance determined by one-way ANOVA followed by Tukey’s multiple comparisons test. $p < 0.0001$ (one-way ANOVA, $F = 45.35$, $R^2 = 0.40$; Tukey's post hoc). Sample sizes (n) are indicated in the figure panels. Data are represented as mean +/- SEM.

(D) MD assays of flies expressing the *empty-GAL4* driver together with *SIFaR-RNAi*. Statistical significance determined by one-way ANOVA followed by Tukey’s multiple comparisons test. $p < 0.0001$ (one-way ANOVA, $F = 42.00$, $R^2 = 0.40$; Tukey's post hoc). Sample sizes (n) are indicated in the figure panels. Data are represented as mean +/- SEM.

Fig.S4 Specific SIFaR-expressing neurons regulate mating duration.

(A) MD assays of flies expressing the empty-*GAL4* driver together with *UAS-stop-TNT; fru*^{FLP}. Statistical significance determined by one-way ANOVA followed by Tukey’s multiple comparisons test. $p < 0.0001$ (one-way ANOVA, $F = 32.70$, $R^2 = 0.34$; Tukey's

post hoc). Sample sizes (n) are indicated in the figure panels. Data are represented as mean +/- SEM.

(B) MD assays of flies expressing the *SIFaR*^{R23G06}*-GAL4* driver together with *UAS-stop-TNT*^{in}*; fru*^{FLP}. Statistical significance determined by one-way ANOVA followed by Tukey's multiple comparisons test. $p < 0.0001$ (one-way ANOVA, $F = 23.86$, $R^2 = 0.22$; Tukey's post hoc). Sample sizes (n) are indicated in the figure panels. Data are represented as mean +/- SEM.

(C) MD assays of flies expressing the *SIFaR*^{R24A12}*-GAL4* driver together with *UAS-stop- TNT*^{in}*; fru*^{FLP}. Statistical significance determined by one-way ANOVA followed by Tukey's multiple comparisons test. $p < 0.0001$ (one-way ANOVA, $F = 27.33$, $R^2 = 0.26$; Tukey's post hoc). Sample sizes (n) are indicated in the figure panels. Data are represented as mean +/- SEM.

(D) MD assays of flies expressing the *SIFaR*^{R24F06}*-GAL4* driver together with *UAS-stop- TNT*^{in}*; fru*^{FLP}. Statistical significance determined by one-way ANOVA followed by Tukey's multiple comparisons test. $p < 0.0001$ (one-way ANOVA, $F = 18.41$, $R^2 = 0.24$; Tukey's post hoc). Sample sizes (n) are indicated in the figure panels. Data are represented as mean +/- SEM.

(E) MD assays of flies expressing the *SIFaR*^{R57F10}*-GAL4* driver together with *UAS-stop- TNT*^{in}*; fru*^{FLP}. Statistical significance determined by one-way ANOVA followed by Tukey's multiple comparisons test. $p < 0.0001$ (one-way ANOVA, $F = 1.014$, $R^2 = 0.37$; Tukey's post hoc). Sample sizes (n) are indicated in the figure panels. Data are represented as mean +/- SEM.

(F) CL of group-housed males expressing *empty-GAL4, SIFaR^{R23G06}-GAL4, SIFaR^{R24A12}-GAL4, SIFaR^{R24F06}-GAL4* and *SIFaR^{R57F10}-GAL4* together with *SIFaR-RNAi*. Statistical significance determined by one-way ANOVA followed by Tukey's multiple comparisons test. $p < 0.0001$ (one-way ANOVA, $F = 3.053$, $R^2 = 0.02$; Tukey's post hoc). Sample sizes (n) are indicated in the figure panels. Data are represented as mean +/- SEM.

(G) Male(upper) and female(below) flies expressing *SIFaR^{R57F10}* and *fru^{FLP}* together with *UAS-RedStinger* and *UAS-stop-mCD8GFP* were immunostained with anti-GFP (green), anti-RFP (red), nc82 (blue) antibodies. The left two panels are presented as a gray scale to clearly show the GFP and RFP signal. Scale bars represent 100 µm.

Fig.S5 SIFaR in Tk neurons specifically regulates copulation latency

(A) Male flies brain expressing *fruP1-GAL4* and *lexA^{24F06}* drivers together with *UAS-mCD8RFP* and *lexAop-mCD8GFP*. Dased circle indicate the region of interest. Scale bars represent 50 µm.

(B) Male flies brain expressing *fruP1-GAL4* and *SIFaR^{T2A}-lexA* drivers together with *UAS-mCD8RFP* and *lexAop-mCD8GFP*. Dased circle indicate the region of interest. Scale bars represent 50 µm.

(C) MD assays of flies expressing the *fruP1-GAL4* driver together with *Tsh-GAL80* and *SIFaR-RNAi*. Statistical significance determined by one-way ANOVA followed by Tukey's multiple comparisons test. $p = 0.9814$ (one-way ANOVA, $F = 0.019$, $R^2 = 0.00049$; Tukey's post hoc). Sample sizes (n) are indicated in the figure panels. Data are

represented as mean +/- SEM.

(D) CI of group-housed males expressing *fruP1-GAL4* together with *Tsh-GAL80* and *SIFaR-RNAi* (two-tailed unpaired *t*-test). In all plots and statistical tests. Data are presented as mean ± s.e.m. ns = not significant ($p>0.05$), *$p<0.05$, **$p<0.01$, ***$p< 0.001$, ****$p< 0.0001$. Sample sizes (n) are indicated in the figure panels. Data are represented as mean +/- SEM.

(E) CL of group-housed males expressing *fruP1-GAL4* together with *Tsh-GAL80* and *SIFaR-RNAi* (two-tailed unpaired *t*-test). In all plots and statistical tests. Data are presented as mean ± s.e.m. ns = not significant ($p>0.05$), *$p<0.05$, **$p<0.01$, ***$p< 0.001$, ****$p< 0.0001$. Sample sizes (n) are indicated in the figure panels. Data are represented as mean +/- SEM.

(F) Male flies brain expressing $GAL4^{R43D01}$ and $lexA^{24F06}$ drivers together with *UAS-mCD8RFP* and *lexAop-mCD8GFP*. Dased circle indicate the region of interest. Scale bars represent 100 μm.

(G) Male flies expressing *fruP1-GAL4* along with *LexAop-CD2-GFP, UAS-mLexA-VP16-NFAT and LexAop-CD8-GFP-A2-CD8-GFP* were dissected after 5 days of growth (mated male flies had 1-day of sexual experience with virgin females). The dissected brains were then immunostained with anti-GFP (green) and anti-nc82 (blue). GFP is pseudo-colored as “red hot”.

(H) MD assays of flies expressing the *Tk-GAL4* driver together with *SIFaR-RNAi*. Statistical significance determined by one-way ANOVA followed by Tukey’s multiple comparisons test. p = 0.0002 (one-way ANOVA, F = 9.001, R^2 = 0.11; Tukey's post

hoc). Sample sizes (n) are indicated in the figure panels. Data are represented as mean +/- SEM.

(I) CL of group-housed males expressing *Tk-GAL4* together with *empty-RNAi* and *SIFaR-RNAi* (two-tailed unpaired *t*-test). In all plots and statistical tests. Data are presented as mean ± s.e.m. ns = not significant ($p>0.05$), *$p<0.05$, **$p<0.01$, ***$p< 0.001$, ****$p< 0.0001$. Sample sizes (n) are indicated in the figure panels. The asterisks (*) indicating statistical significance are marked in pink, representing that the CL of this genotype was significantly longer than that of control group. Data are represented as mean +/- SEM.

(J) MD assays of flies expressing the *SIFaR-GAL4* driver together with *Tk-RNAi*. Statistical significance determined by one-way ANOVA followed by Tukey’s multiple comparisons test. $p < 0.0001$ (one-way ANOVA, $F = 16.20$, $R^2 = 0.093$; Tukey's post hoc). Sample sizes (n) are indicated in the figure panels. Data are represented as mean +/- SEM.

(K) CL of group-housed males expressing *empty-GAL4* and *SIFaR-GAL4* together with *Tk-RNAi* (two-tailed unpaired *t*-test). In all plots and statistical tests. Data are presented as mean ± s.e.m. ns = not significant ($p>0.05$), *$p<0.05$, **$p<0.01$, ***$p< 0.001$, ****$p< 0.0001$. Sample sizes (n) are indicated in the figure panels. The asterisks (*) indicating statistical significance are marked in pink, representing that the CL of this genotype was significantly longer than that of control group. Data are represented as mean +/- SEM.

Fig.S6 SIFaR expression in mAL neurons creates context-specific internal states through synaptic plasticity.

(A) Male flies expressing *GAL4*^{R43D01} and *SIFaR*^{2A}*-lexA* together with *UAS-Stinger* and *LexAop-tdTomato.nls* were immunostained with anti-GFP (green), anti-RFP (red), nc82 (blue) antibodies. Scale bars represent 100 μm. Arrows indicate the cells which labelled by *GAL4*^{R43D01} and *SIFaR*^{2A}*-lexA*.

(B) Male flies expressing *GAL4*$^{SIFa.PT}$ and *lexA*$^{SIFa.PT}$ together with *UAS-mCD8RFP* and *LexAop-mCD8GFP* were immunostained with anti-GFP (green), anti-RFP (red), nc82 (blue) antibodies. Scale bars represent 100 μm. Arrows indicate the cells which labelled by *GAL4*$^{SIFa.PT}$ and *lexA*$^{SIFa.PT}$.

(C) Diagram of how SIFa orchestrate mating investment through SIFaR in mAL and fruP1 neurons.

Supporting information. 1

(A) Fly cell atlas data showing the expression pattern of NPs and NPRs across different cell types. The scaled normalized UMIs are indicated by the intensity of the dot color (Average Expression).
